# Host genotype-specific rhizosphere fungus enhances drought resistance in wheat

**DOI:** 10.1186/s40168-024-01770-8

**Published:** 2024-03-04

**Authors:** Hong Yue, Xuming Sun, Tingting Wang, Ali Zhang, Dejun Han, Gehong Wei, Weining Song, Duntao Shu

**Affiliations:** 1 College of Agronomy, National Key Laboratory of Crop Improvement for Stress Tolerance and Production, Northwest A&F University, Yangling, Shaanxi 712100 China; 2 College of Life Sciences, National Key Laboratory of Crop Improvement for Stress Tolerance and Production, Northwest A&F University, Yangling, Shaanxi 712100 China; 3 Shaanxi Key Laboratory of Agricultural and Environmental Microbiology, Yangling, Shaanxi 712100 China

**Keywords:** Wheat, Rhizosphere, Microbiome, Drought, Multi-omics

## Abstract

**Background:**

The severity and frequency of drought are expected to increase substantially in the coming century and dramatically reduce crop yields. Manipulation of rhizosphere microbiomes is an emerging strategy for mitigating drought stress in agroecosystems. However, little is known about the mechanisms underlying how drought-resistant plant recruitment of specific rhizosphere fungi enhances drought adaptation of drought-sensitive wheats. Here, we investigated microbial community assembly features and functional profiles of rhizosphere microbiomes related to drought-resistant and drought-sensitive wheats by amplicon and shotgun metagenome sequencing techniques. We then established evident linkages between root morphology traits and putative keystone taxa based on microbial inoculation experiments. Furthermore, root RNA sequencing and RT-qPCR were employed to explore the mechanisms how rhizosphere microbes modify plant response traits to drought stresses.

**Results:**

Our results indicated that host plant signature, plant niche compartment, and planting site jointly contribute to the variation of soil microbiome assembly and functional adaptation, with a relatively greater effect of host plant signature observed for the rhizosphere fungi community. Importantly, drought-resistant wheat (Yunhan 618) possessed more diverse bacterial and fungal taxa than that of the drought-sensitive wheat (Chinese Spring), particularly for specific fungal species. In terms of microbial interkingdom association networks, the drought-resistant variety possessed more complex microbial networks. Metagenomics analyses further suggested that the enriched rhizosphere microbiomes belonging to the drought-resistant cultivar had a higher investment in energy metabolism, particularly in carbon cycling, that shaped their distinctive drought tolerance via the mediation of drought-induced feedback functional pathways. Furthermore, we observed that host plant signature drives the differentiation in the ecological role of the cultivable fungal species *Mortierella alpine* (*M*. *alpina*) and *Epicoccum nigrum* (*E. nigrum*). The successful colonization of *M*. *alpina* on the root surface enhanced the resistance of wheats in response to drought stresses via activation of drought-responsive genes (e.g., *CIPK9* and *PP2C30*). Notably, we found that lateral roots and root hairs were significantly suppressed by co-colonization of a drought-enriched fungus (*M*. *alpina*) and a drought-depleted fungus (*E. nigrum*).

**Conclusions:**

Collectively, our findings revealed host genotypes profoundly influence rhizosphere microbiome assembly and functional adaptation, as well as it provides evidence that drought-resistant plant recruitment of specific rhizosphere fungi enhances drought tolerance of drought-sensitive wheats. These findings significantly underpin our understanding of the complex feedbacks between plants and microbes during drought, and lay a foundation for steering “beneficial keystone biome” to develop more resilient and productive crops under climate change.

Video Abstract

**Supplementary Information:**

The online version contains supplementary material available at 10.1186/s40168-024-01770-8.

## Background

Drought is one of the most significant obstacles to agricultural productivity worldwide [12, 22, 72]. The frequency and severity of drought stress are yearly increasing as a result from global climate change and human activities in the current century [4, 75]. Wheat (*Triticum aestivum* L.) is a major food staple that contributes more than 20% of global caloric intake [2]. Although a wide range of wheat varieties are cultivated worldwide, their average annual production has been reduced by 58–72% when confronting drought stress during their growth stages [68]. Therefore, there is an urgent need to enhance the drought tolerance of wheat, which is essential for improving crop production and global food security [16].

Plants and their associated microbes have coevolved for more than 450 million years and formed a “holobiont” [14, 28], whereby microbes usually act as game-changers and are facilitators of ecosystem change [13]. More importantly, these microbes play critical roles in maintaining ecosystem stability, including disease suppression, nutrient acquisition, and tolerance of abiotic stress [31, 39]. An effective and environmentally sustainable approach for enhancing plant resistance and mitigating the impacts of drought stress on crop yield is via the manipulation of the plant-associated microbiomes [73, 74]. Pioneering studies highlights rhizosphere microbiomes can also regulate the physiological responses of host plants to drought stress and enhance plant adaptation under global climate change scenarios [51, 57, 63]. However, prior to being able to manipulate rhizosphere-associated microbiomes for plant drought resistance, we must understand how the drought response traits of microbes confer drought tolerance on host plants.

The host plant genotype is the key factor that shapes the rhizosphere microbiome assemblage [46], in particular for individual microbial recruitment processes [6]. A pioneer study of the influence of drought stress on a sorghum plant-associated microbiome in a field experiment revealed that the host genotype exerted a significant selective effect on these microbes [24]. Moreover, genetic aspects of the host had distinct effects on the sorghum mycobiome and arbuscular mycorrhizal fungi when the host plant suffered from drought stress [23]. Given that different groups of rhizosphere microbes have different degrees of drought tolerance, certain bacteria and fungi can be utilized by distinctive genotypes of host plants via the release of specific root exudates, which is known as the “cry for help” hypothesis [3]. During these complex plant–microbiome interactions, more drought-resistant taxa may be recruited by plants and are in turn able to enhance the host plants’ responses to drought [29]. Prior work based on genome-resolved metagenomics revealed that the activation of microbial iron metabolism and glycerol-3-phosphate uptake led to a significant increase in the drought-induced enrichment of Actinobacteria, as well as their promotion in the host genotype during drought stress [73]. These pioneer efforts provided insights into microbial drought tolerance mechanisms and made it possible to precisely manipulate genotype-specific microbes to enhance plant growth under drought stress. However, more molecular evidence and a precise analysis of how microbial traits modify plant responses to drought have been hampered by a lack of functional and genetic details associated with drought stress for distinctive genotypes of plants, which would allow a comparative analysis between the responses of drought-resistant and drought-sensitive cultivars.

In the present study, we postulated that drought-enriched genotype-specific microbes may be more capable of enhancing drought-responsive traits of host plants and thus enhancing the drought resistance of drought-sensitive cultivars. Verifying this hypothesis could pave the way for manipulation of rhizosphere microbiomes to maintain plant performance in response to drought stress. Specifically, our aims were to (1) reveal which rhizosphere microbiomes are significantly affected by plant genotypes, as well as the complexity of microbial interkingdom association networks in different drought-resistant varieties; (2) decipher how drought-enriched and drought-depleted microbes affect plant morphological traits; and (3) elucidate the mechanisms whereby drought-enriched rhizosphere microbes promote drought resistance in wheat.

## Materials and methods

### Field experiment design and sample collection

Long-term field experiments were established with typical winter wheat–summer fallow crop rotations since November 2007. Two wheat varieties, namely, Chinese Spring (CS) and Yunhan 618 (YH), were employed in this study. CS wheat is generally considered to be the standard cultivar for wheat research and is highly sensitive to drought stress, whereas YH wheat is a well-known drought-resistant genotype that has stronger resistance to drought than CS wheat. Given that the geographic location and associated soil type play an important role in shaping plant-associated microbial community assemblages, we therefore conducted field experiments in two geographically distant fields, namely, Yangling (YL, 108° 6′ E, 34° 18′ N) and Suqian (SQ, 118° 19′ E, 34° 1′ N). The historical monthly average rainfall at the YL site, which is located in a semi-arid area, was 12 mm, whereas the SQ site is located in a semi-humid area with a monthly average rainfall of 39 mm (Table S1). In terms of the field layout, the CS and YH wheat varieties were planted in a randomized block design that considered genotype and replication, with five replicate blocks. All blocks at these two sites were fertilized with inorganic fertilizers comprising urea (120 kg N ha^−1^ year^−1^), diammonium phosphate (300 kg P_2_O_5_ ha^−1^ year^−1^), and potassium chloride (150 kg KCl ha^−1^ year^−1^). No irrigation, herbicides, or pesticides were applied during the whole growth period.

Given that the jointing stage in plants represents relative sensitivity of soil microbiomes to water limitations, drought tolerance-associated rhizosphere microbes may be significantly enriched in drought-resistant varieties. We therefore collected soil samples from two sites during the jointing stage of wheat (April 18, 2019 for YL site and March 25, 2019 for SQ site). In brief, around 30 individual wheat plants from each block were randomly selected, and rhizosphere soil samples that firmly adhered to the roots were carefully collected by gently brushing the roots. Moreover, samples of topsoil (5–20 cm) that was around 20 cm away from the plants horizontally were collected as bulk soil using an auger corer. The obtained soil samples were passed through sterilized 2-mm meshes to remove straw residues and roots, which generated a total of 40 soil samples for downstream analysis (5 replicates × 2 varieties × 2 sites × 2 compartments).

### DNA extraction and amplification sequencing

Extraction of total DNA from the collected rhizosphere and bulk soil samples was performed using a FastDNA SPIN Kit for Soil (MP Biomedicals, USA) according to the manufacturer’s instructions. The V4–V5 region of the bacterial 16S rRNA gene was amplified using the primers 515F (5′-GTGYCAGCMGCCGCGGTAA-3′) and 907R (5′-CCGTCAATTCMTTTRAGTTT-3′). The primer pairs used for targeting the fungal ITS1 region were ITS1F (5′-CTTGGTCATTTAGAGGAAGTAA-3′) and ITS2R (5′-GCTGCGTTCTTCATCGATGC-3′). The PCR kit included 4 μL 5 × FastPfu buffer, 2 μL dNTP (2.5 mM), 10 ng DNA, 0.8 μL forward and reverse primers (5 μM), and 0.4 μL FastPfu polymerase. The PCR for each sample was conducted in triplicate to reduce bias, and the PCR products were purified with an AxyPrep DNA Gel Extraction Kit (Axygen Biosciences, USA). After quantification using a Qubit 3 Fluorometer, the triplicate products were pooled in equimolar concentrations. Prior to sequencing, the generated libraries were cleaned using Beckman AMPure XP beads (Beckman Coulter, U.S.A), and they were then sequenced on the Illumina MiSeq platform using the PE 2 × 300 approach (Shanghai Majorbio Bio-Pharm Technology Co., Ltd, China). For sequence processing, all the generated raw reads were analyzed using the QIIME 2 (2022.7; https://qiime2.org)-CentOS7.6 environment. The q2-vesearch plugin in QIIME 2 was used to demultiplex, quality control, and cluster raw sequences [5]. The obtained operational taxonomic units (OTUs) were assigned to taxonomic classifications with the SILVA database (release 138) for bacteria and UNITE database (version 7.2) for fungi. The OTU feature table was rarified to the same sequencing depth per sample for downstream analysis (40,580 for bacteria and 30,355 for fungi).

### Shotgun metagenomics sequencing and functional gene annotation

Given the significant differences of dominant fungi phyla in the plant rhizosphere between drought-resistant and drought-sensitive wheats, shotgun metagenomic analyses of 12 libraries (2 varieties × 2 sites × 3 replicates) were performed on the extracted rhizosphere soil DNA using 150 bp paired-end read sequencing run on a HiSeq 2500 sequencer (Illumina, USA). The raw reads obtained from all samples were subjected to quality control using fastp [10]. The obtained clean reads were assembled using IDBA-UD [50] and were then clustered with a similarity threshold of 0.90 using CD-HIT to generate a non-redundant gene catalog [21]. Lengths of contigs that were greater than 500 bp were used to perform open reading frame prediction with MetaGene [47]. Relative abundances between different samples for high-quality reads using the criterion “identity > 95%” were calculated by SOAPaligner [26]. All high-quality contigs were clustered into different pathways on the basis of BLASTP queries against the Kyoto Encyclopedia of Genes and Genomes (KEGG) pathway database with an e-value of < 1e − 5. Functional profiles, including carbohydrate-active enzymes (CAZyome), of rhizosphere microbiomes were determined using the CAZy database. Orthologous groups of proteins (COG) were identified using the eggNOG database (release 5.0), and KEGG Orthology (KO) enrichment was performed using the KEGG database (release 80.1) [33]. The “phyper” function in the R “stats” package was used to determine the enrichment status of KEGG pathways. The Shannon index was calculated and principal component analysis (PCoA) of functional genes was performed using the R “vegan” package [48]. Raw metagenomics datasets have been deposited in NCBI SRA database.

### Rhizosphere microbiota isolation, inoculation, and synthetic community construction

**Fig. 1:**
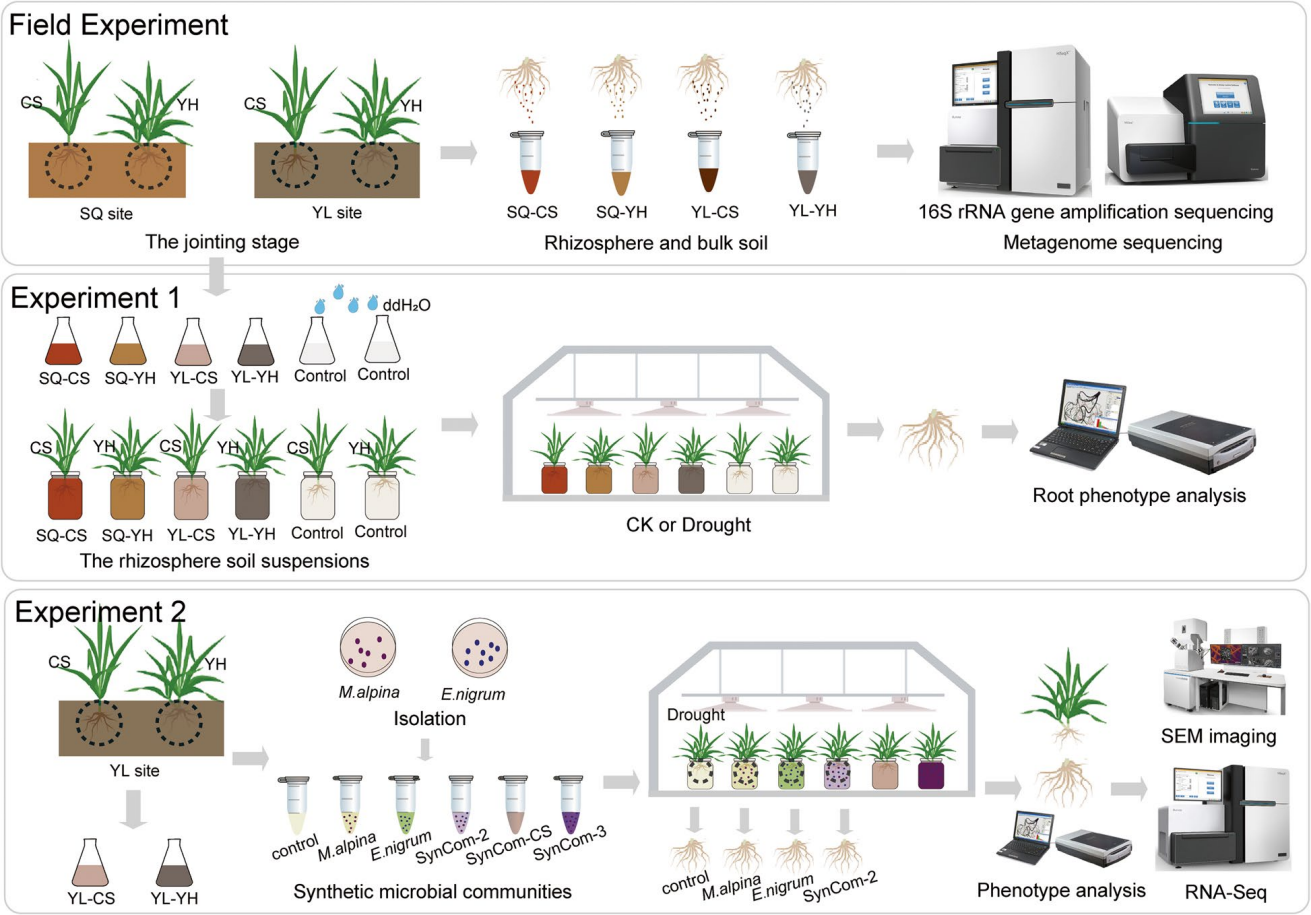
Experimental flow charts for this study. **a** Long-term field experiments were established starting in 2007. Two wheat varieties, namely, Chinese Spring (CS) and Yunhan 618 (YH), were employed in this study. Bulk and rhizosphere soils were sampled during the jointing stage for amplicon and metagenomics sequencing. **b** Procedure for Experiment 1, the rhizosphere soil samples were used to generate microbial suspension cultures to conduct pot experiment. After inoculation, the root samples from different groups were collected for root morphology trait analysis. **c** Procedure for Experiment 2, isolation of rhizosphere fungi from Experiment 1 and construction of SynComs to confirm the ecological role of specific rhizosphere fungi recruited by plants in enhancing plant drought tolerance. Root-related parameters and plant gene expression were conducted by using phenotype analysis, SEM imaging, and RAN-Seq techniques when the roots were harvested after inoculation

We conducted two independent experiments to establish whether drought-enriched rhizosphere microbes improve the drought tolerance of wheat (Fig. 1).

In Experiment 1, we aimed to examine whether drought-enriched rhizosphere microbial communities play a pivotal role in enhancing plant growth in the drought-sensitive variety under drought stress. We conducted a three-level factorial experiment (drought treatment × plant variety × rhizosphere microbiota type) to investigate whether rhizosphere microbes (control vs. polyethylene glycol [PEG 6000]-treated) could influence morphological traits of the YH and CS varieties from the YL and SQ sites in response to drought stress. The drought treatments comprised treatment with (i) 0% PEG 6000 and (ii) 10% PEG 6000 [40, 67]. The plant varieties comprised drought-resistant (YH) and drought-sensitive (CS) wheat plants, and rhizosphere microbiota were generated from soils from the YL and SQ planting areas. Together, this resulted in a total of 48 experimental treatments (2 drought treatments × 2 plant varieties × 2 rhizosphere microbiota types × 6 replicates). For the generation of rhizosphere microbiota, 5 g rhizosphere soil was shaken with 50 mL nutrient-rich medium (tryptic soy broth) at 60 rpm for 2 days. The supernatants were then collected and shaken for 3 days at 28 °C. The generated suspensions of rhizosphere microbiota were stored at 4 °C and it were then used to soaking plant roots. Prior to inoculation, surface-sterilized and germinated seeds were grown in Hoagland nutrient solution at 27 °C. During inoculation, the nutrient solution used for each treatment was refreshed at intervals of 4 days. After inoculation with rhizosphere microbiota for 2 weeks, plant roots were carefully washed with ddH_2_O, and root-related parameters include taproots and lateral roots were measured using an LA-S Plant Root Analysis System (Wan Shen, Hangzhou, China).

In Experiment 2, we aimed to examine whether specific rhizosphere fungi recruited by plants under drought stress were capable of enhancing plant drought tolerance. We conducted a two-level factorial experiment (plant variety × inoculation microbiota type) to evaluate the effects of single fungi strains or synthetic community (SynCom) on plant drought tolerance. The CS wheat plants were used as the plant variety, and there were six replicates for each treatment. Given the significant associations between root morphological traits and drought-enriched fungi, the drought-enriched fungus *Mortierella alpina* and the drought-depleted fungus *Epicoccum nigrum* were selected to conduct a follow-up experiment. The treatments in terms of inoculation microbiota type were as follows: (i) control without microbiota inoculation, (ii) *M. alpina*, (iii) *E. nigrum*, (iv) SynCom-2, (v) SynCom-CS, and (vi) SynCom-3. To isolate the two fungal strains, *M. alpina* and *E. nigrum* were grown on solid potato dextrose agar (PDA) medium and incubated at 28 and 25 °C, respectively. After 2 weeks, 100 mg fungus was harvested from the solid PDA medium, transferred into 1 mL MgCl_2_ with sterile stainless-steel beads, and crushed with a paint shaker to obtain mycelial fragments, as previously described [30]. After that, 1 mL of the fungal suspension was added to 100 mL Hoagland nutrient solution to form a suspension of a single fungal strain. We constructed SynCom-2, which consisted of *M. alpina* and *E. nigrum*, by mixing equal volumes of the suspensions of both species. SynCom-CS was generated by mixing 1 mL of the rhizosphere microbiota suspension from CS wheat with 100 mL Hoagland nutrient solution. SynCom-3 contained *M. alpina*, *E. nigrum*, and SynCom-CS and was generated by mixing equal volumes of the different suspensions. The control group received 1 mL MgCl_2_ instead of a fungal suspension. Plant roots were soaked with SynComs using similar approaches to those mentioned in Experiment 1. After 18 days, root-related parameters, fresh weights of shoots, and the dry weight of each seedling were measured to assess plant drought resistance.

### Scanning electron microscopy (SEM) imaging

Prior to imaging, wheat roots inoculated with *M. alpina*, *E. nigrum*, and SynComs were carefully washed three times with dd H_2_O to remove loosely attached microorganisms. The apical 5-mm segments of root ends were sectioned using a Leica VT1000 S vibratome (Leica, Nussloch, Germany) and were fixed at 4 °C for 12 h using 4% glutaraldehyde (v/v). After the fixing period, the root samples were washed four times with 0.1 M PBS buffer (pH 6.8), and then a graded ethanol series (30–100%, v/v) was used for dehydration. Then, root samples subjected to each treatment were observed under a field-emission scanning electron microscope (Nova NanoSEM 450, Oregon, USA).

### RNA sequencing and quantitative real-time PCR

Roots of 18-day-old CS with or without colonization by SynComs and subjected to drought stress were harvested for RNA-Seq and quantitative real-time PCR (qRT-PCR) analysis. Total RNA was extracted with an RNAsimple Total RNA Kit (Tiangen, Beijing, China). RNA-Seq libraries were prepared and sequenced on the Illumina 2000 platform (Majorbio Co., Ltd, Shanghai, China). Raw reads were checked and cleaned using fastp (release 0.23.2). The cleaned reads were mapped to the annotated genome of wheat (IWGSC v2.1) using TopHat2 (v.2.1.1), and the transcript expression of genes was calculated using Salmon (https://combine-lab.github.io/salmon/). Log_2_ fold changes (log_2_FC) and *P*-values for gene expression were calculated using the edgeR package. When |log_2_FC|> 1 with a false discovery rate (FDR) of ≤ 0.05, this was considered to represent a significant difference between the control and treatment groups. Gene Ontology enrichment analysis was carried out using the BiNGO plugin in Cytoscape (release 3.9.1) [62], and KEGG enrichment analysis was implemented using the clusterProfiler package [70]. Overlapping differentially expressed genes (DEGs) in all groups were subjected to *k*-means clustering. Three biological replicates from each sample were selected to conduct the abovementioned transcriptome analyses.

Prior to qRT-PCR, 1 μg RNA from each sample was used for cDNA synthesis using a PrimeScript 1st strand cDNA Synthesis Kit (Takara, Japan) according to the manufacturer’s instructions. Then, a total of 15 DEGs associated with responses to drought stress were further selected for qRT-PCR using an SYBR Green Premix Pro Taq HS qPCR Kit (AG11718, Accurate Biotechnology (Hunan), China) on a StepOnePlus Real-Time PCR System (ABI, USA). The housekeeping gene β-actin was used as the reference gene for all qRT-PCR reactions. The 2^−ΔΔCT^ method was used to calculate the relative expression levels of the target genes. The primers used here are summarized in the supplementary data (Table S2).

## Results

### Drought-induced shifts in rhizosphere microbiomes associated with plant genotypes

**Fig. 2:**
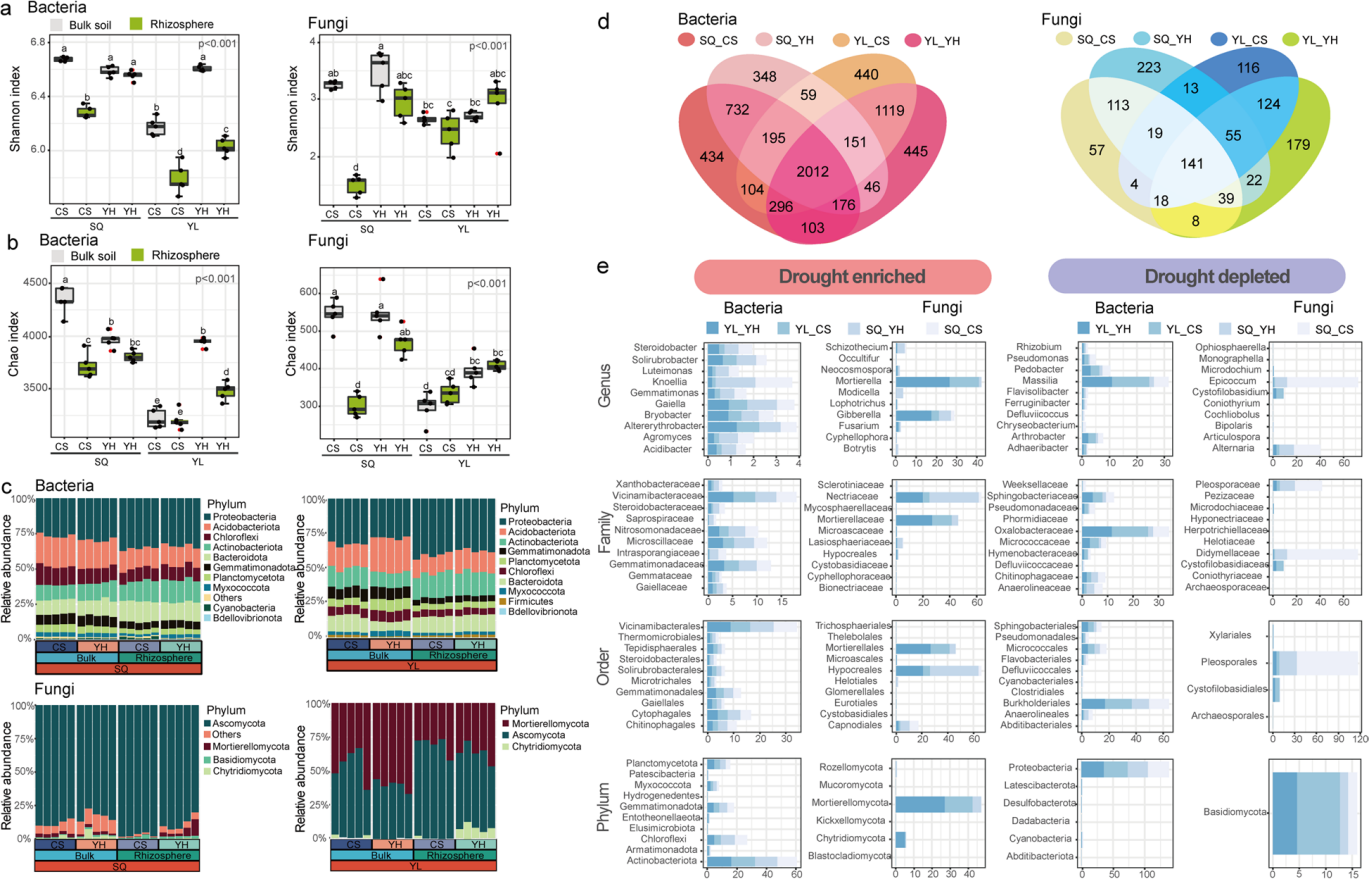
Patterns of diversity of soil microbiomes and influences of drought stress on enriched bacterial and fungal taxa. **a** and **b** represent the Shannon and Chao indices of bacterial and fungal communities, respectively, in all samples. A difference in the letters indicates a significant difference (*P* < 0.05, *n* = 5). Abbreviations: Suqian site (SQ), Yangling site (YL), drought-susceptible wheat cultivar Chinese Spring (CS), drought-resistant wheat cultivar Yunhan 618 (YH), bulk soil (B), and rhizosphere soil (T). **c** Relative abundance of dominant phylum in different wheat cultivars at SQ (left panel) and YL (right panel) sites. **d** Shared operational taxonomic units (OTUs) in drought-resistant and drought-susceptible cultivars across the YL and SQ sites. **e** Relative abundances of drought-enriched and drought-depleted OTUs in bacteria and fungi at genus, family, order, and phylum levels

We first compared the community diversity of bulk soil and rhizosphere microbiomes between drought-resistant (YH) and drought-susceptible (CS) varieties at the SQ and YL planting sites. The Shannon index and Chao1 richness of the rhizosphere bacterial communities of the YH cultivar at the SQ and YL sites were significantly higher than those of the CS cultivar at both planting sites (Fig. 2a, b). The variability of the rhizosphere fungal communities in terms of community diversity and richness exhibited consistent patterns (Fig. 2a, b). These results indicated that the rhizosphere microbiome in the drought-resistant cultivar possessed higher community diversity than that of the drought-sensitive cultivar, regardless of the planting area. Regarding the variability of the microbial communities at the YL planting site, the PERMANOVA analyses further revealed that the greatest effect on the soil microbiomes was exerted by the compartment (*R*^2^ = 0.457 for bacteria and *R*^2^ = 0.470 for fungi; *P* < 0.001), followed by the plant genotype (*R*^2^ = 0.172 for bacteria and *R*^2^ = 0.109 for fungi;* P* < 0.001; Table S3). Similarly, the plant genotype also explained a relatively high proportion of the variation in the total microbiomes at the SQ planting site (*R*^2^ = 0.135 for bacteria and *R*^2^ = 0.136 for fungi; *P* < 0.001; Table S3). The CAP and PCoA analyses also revealed that host plant signature, plant niche compartment, and planting site jointly had effects on the variation of soil bacterial and fungal communities (Permutest, *P* = 0.001; Figs. S1 and S2). These results indicated that the drought-resistant wheat harbored more diverse bacterial and fungal taxa than the drought-sensitive wheat.

Considering the greater discrepancy of local climate (e.g., mean annual precipitation) between YL and SQ sites, we observed that planting sites had a greater influence on the relative abundance of dominant bacteria and fungi phyla than those in YH and CS wheats (Fig. 2c). Although there is a leaping change in bacterial and fungal abundance in both two planting sites, we further found that bacterial phyla, including Actinobacteria, Acidobacteriota, Proteobacteria, and Bacteroidota, exhibited distinct enrichment patterns. More importantly, the fungal phyla Mortierellomycota and Chytridiomycota exhibited significantly contrasting patterns in YH and CS wheats, respectively (Fig. 2c; Fig. S3; Tables S4 and S5).

Moreover, rhizosphere microbiomes in the drought-resistant and drought-susceptible varieties formed distinctive ecological clusters (Fig. 2d). Based on the drought-enriched and drought-depleted OTUs (Figs. S4 and S5), we further confirmed that 46 bacterial OTUs and 36 fungal OTUs were considered as drought-responsive OTUs at the genus level. As expected, these drought-responsive OTUs also present significant difference in the abundance at both planting areas (*P* < 0.05). We further found that the drought-enriched OTUs included the bacterial genera *Massilia* and *Pedobacter* and the fungal genera *Mortierella* and *Gibberella*. As for drought-depleted OTUs, the bacterial genera *Ramlibacter* and *Knoellia* and the fungal genera *Epicoccum* and *Fusicolla* were confirmed using enrichment analyses (Fig. 2e; Fig. S6; Tables S6 and S7). These results suggested that distinctive patterns of drought-responsive taxa were closely associated with plant genotypes. In addition, by blasting with the plant beneficial and pathogenic microbes database [37], we identified 24 plant pathogen fungi across all the soil samples. For these pathogen fungi, the dominant fungi phyla were Ascomycota (22 genus), following by Basidiomycota (1 genus), Blastocladiomycota (1 genera), and Chytridiomycota (1 genera). The relative abundance of Ascomycota in the SQ site (33.0–33.4% of total ASVs) were significantly higher than that in the YL site (33.0–33.4% of total ASVs), regardless of plant varieties (Table S8 and Fig. S7).

### The drought-resistant variety possessed more complex rhizosphere microbial interkingdom interaction networks

**Fig. 3:**
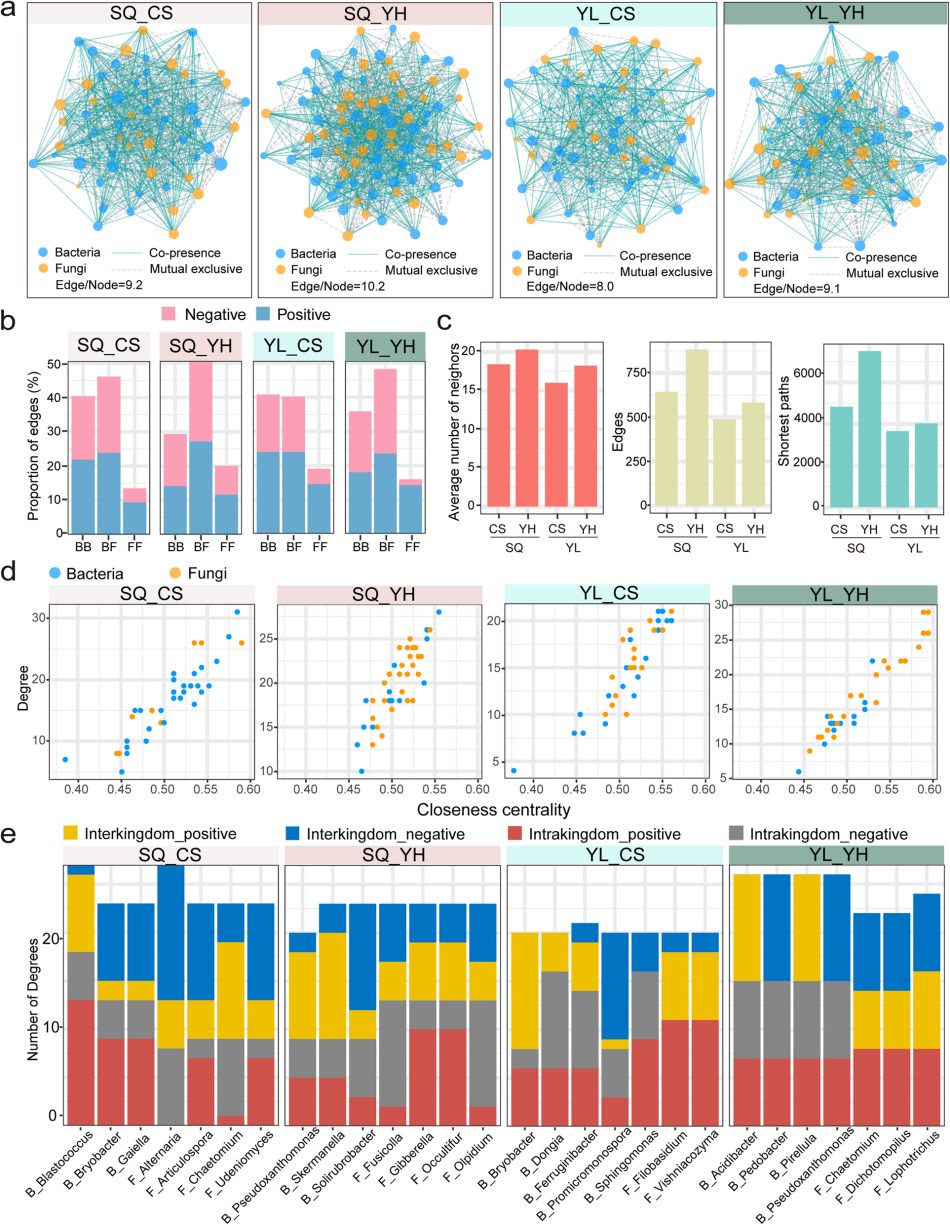
Microbial interkingdom association networks under drought stress. **a** Interkingdom co-occurrence networks of drought-resistant and drought-susceptible cultivars at YL and SQ sites. Only compositionality-robust (|*ρ*|> 0.8) and statistically significant (*P* < 0.05) correlations are shown. The size of each node indicates the relative abundance of the corresponding amplicon sequence variant (ASV). Blue solid lines represent co-presence associations, and gray dotted lines represent mutual exclusion correlations. The thickness of each line is proportional to the correlation coefficient of the corresponding association. **b** Bar graph showing the proportion of edges in bacterial–bacterial (BB), bacterial–fungal (BF), and fungal–fungal (FF) correlations in the different samples. **c** Average numbers of neighbors, edges, and shortest paths in the different samples. **d** Scatter plot showing the features of degree and closeness centrality for the different wheat cultivars. **e** Degree and interaction type of the top seven hub nodes in the drought-resistant and drought-susceptible cultivars

To explore how the difference in the drought resistance of the varieties affected rhizosphere microbiome co-occurrence profiles, we constructed bacterial–fungal interkingdom interaction networks using significant pairwise correlations. For the SQ planting site, we observed that the rhizosphere microbiome in the drought-resistant cultivar had a higher number of instances of co-occurrence (edge/node ratio = 10.2 in YH wheat) than the drought-susceptible variety (edge/node ratio = 9.2 in CS wheat). We also recorded a higher proportion of instances of microbial co-occurrence in YH wheat (edge/node ratio = 9.1) than in CS wheat (edge/node ratio = 8.0) at the YL planting area (Fig. 3a). We then inspected the proportions of negative and positive correlations in the four networks and found that negative bacterial–fungal interkingdom correlations in YH wheat at both planting sites were stronger than those in CS wheat (Fig. 3b). Interestingly, positive correlations dominated fungal–fungal intrakingdom correlations in all networks. The higher network connectedness and more negative bacterial–fungal correlations indicated that the drought-resistant variety had more complex rhizosphere microbial interkingdom networks. We then turned our attention to topological features of the networks and found that node connectedness, the number of shortest paths, and the average number of neighbors were higher in YH wheat than in CS wheat, regardless of the planting site (Fig. 3c; Table S9).

On further inspection of fungal and bacterial OTUs that had high values of closeness centrality and degree in the networks, we found that bacterial taxa accounted for a high proportion of hub nodes in CS wheat (Fig. 3d). In contrast, fungal taxa accounted for a higher proportion of hub nodes in YH wheat. For instance, we detected 10 network hubs (bacteria 7, fungi 3) and 21 network hubs (bacteria 5, fungi 16) in the rhizosphere of CS and YH wheats, respectively, at the SQ site. For the YL site, 7 network hubs (bacteria 5, fungi 2) and 11 network hubs (bacteria 1, fungi 10) were detected in CS and YH wheats, respectively. More importantly, the interkingdom (average proportion of negative edges: 22.2% in the SQ site and 31.4% in the YL site) and intrakingdom correlations (average proportion of negative edges: 23.2% in the SQ site and 28.2% in the YL site) were primarily negative in YH wheat, whereas positive correlations dominated the interkingdom (average proportion of positive edges: 20.5% in the SQ site and 34.7% in the YL site) and intrakingdom associations (average proportion of positive edges: 31.9% in the SQ site and 40.8% in the YL site) in CS wheat (Fig. 3b, e). Given the negative correlations between bacterial and fungal taxa, we safely conclude that these bacterial and fungal OTUs, including *Bacillus*, *Streptomyces*, and *Mortierella*, could act as potential “keystone taxa” in heightening the drought resistant for drought-resistant variety.

### Contrasting patterns of metabolic functions between drought-resistant and drought-sensitive varieties

We then turned our attention to elucidating which specific functional profiles of rhizosphere microbiomes were related to drought responses via a metagenomic sequencing approach (Table S10). According to the results of PCoA (Fig. S8), the functional profiles in terms of KO, COG, and CAZyome in YH wheat revealed a significant separation in comparison with CS wheat at the YL site. We further found that both YH and CS wheats possessed significantly higher functional diversity at the YL site than at the SQ site, in particular in terms of COG and CAZyome diversity (Fig. S9). These results indicated that the drought-resistant and drought-sensitive varieties displayed divergent patterns of metabolic functions, and drought stress increased the functional diversity of specific functional categories.

To further explore the pathways in which all differential KO functional categories were included, KO functional categories enriched in YH and CS wheats that planted in the YL and SQ sites were separately analyzed for KEGG pathway enrichment (Fig. S10). We found that “Amino acid metabolism”, “Carbohydrate metabolism”, and “Energy metabolism” functional categories were enriched in YH wheat, whereas several pathways related to “Membrane transport”, “Signal transduction”, and “Cell growth and death” were enriched in CS wheat at the YL site (Figs. S11 and S12). In particular, “Carbon metabolism” (ko01200) and “Purine metabolism” (ko00230) were found to be significantly enriched in YH wheat at the YL and SQ sites. Concomitantly, “ABC transporters” (ko02010) and “Two-component system” (ko02020) exhibited patterns of significant enrichment in CS wheat at both sites (Fig. S12). Interestingly, the “Carbon fixation” and “Fatty acid degradation” pathways were significantly enriched in YH wheat, regardless of the planting site. Furthermore, several genes associated with drought stress responses displayed a genotype-specific pattern between YH and CS wheats at both planting sites. Specifically, *PsbA*, *chiH*, *PetA*, and *PsbC* genes exhibited significantly differences between CS and YH wheats in the SQ site. For YL site, we found that *SmtA* and *PsbC* showed significantly differences between CS and YH wheats, and *PsbC* were enriched in the YH wheat (Fig. S13). Taken together, site-by-site comparison of functional profiles of rhizosphere microbiomes between different drought-resistant varieties indicated that the enriched rhizosphere microbiomes belonging to the drought-resistant cultivar had a higher investment in energy metabolism, particularly in carbon cycling, that shaped their distinctive drought tolerance via the mediation of drought-induced feedback functional pathways.

### Drought-resistant varieties induced rhizosphere microbiota affects plant growth

**Fig. 4:**
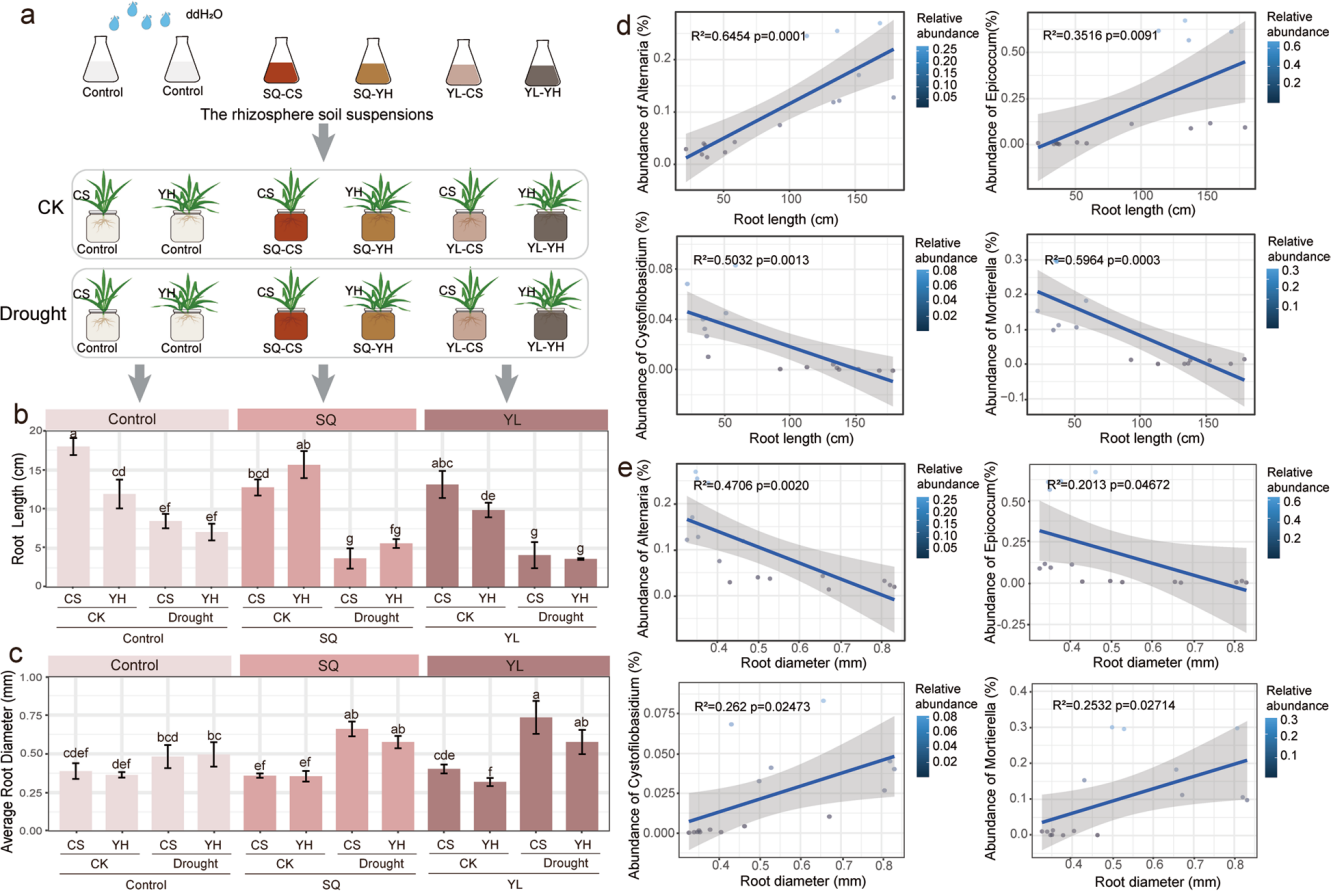
Root morphological traits after different microbial inoculation treatments. Schematic representation of the inoculation experiment. **a** Five-day-old seedlings were grown in stress-free conditions (CK) or under drought stress (polyethylene glycol [PEG 6000]) and were then either not inoculated (Control) or inoculated with rhizosphere microbiota from the YL or SQ site. **b**, **c** The bar graph shows the root length (**b**) and root diameter (**c**) of 14-day-old wheat seedlings grown in the control and SQ and YL rhizosphere microbiota groups, respectively. A difference in the lowercase letters indicates a significant difference at a probability level of *P* < 0.05. **d**, **e** Linear regression relationships between the relative abundance of genotype-specific keystone taxa and root length (**d**) or root diameter (**e**) after the inoculation treatments. The color of the blue dots indicates the relative abundance of the different species

We established a three-level factorial experiment to determine whether the rhizosphere microbial consortium alleviated drought stress and promoted plant growth in the drought-sensitive variety. In the control group, both the root length and the average root diameter of CS wheat were significantly different in comparison with the group inoculated with rhizosphere microbiota from the SQ and YL sites after treatment with 10% PEG 6000 (Fig. 4a–c; Table S11). Importantly, we found that the average root diameter of CS and YH wheats were significantly increased by inoculation with rhizosphere microbiota from the YL site under drought conditions (Fig. 4b) in comparison with the control group. Moreover, the CS wheat exhibited relatively greater root diameter than that of the YH wheat under drought stresses. These results indicated that drought-induced rhizosphere microbiota promotes plant growth under drought conditions via mediating the root morphology traits, and these rhizosphere microbiota has a greater promotion effect on the growth of drought-sensitive wheats.

We further used a correlation-based approach to identify which potential keystone taxa determined root morphological traits. Our results revealed that bacterial OTUs assigned to the phyla Bacteroidota, Proteobacteria, Gemmatimonadota, and Acidobacteriota exhibited significant associations with root morphological traits, such as root length, root surface area, and root diameter (Fig. S14; Table S12). Regarding the fungal community, the genera *Alternaria*, *Cystofilobasidium*, *Mortierella*, and *Epicoccum* displayed significant associations with root length (Fig. 4d) and root diameter (Fig. 4e). More importantly, we were delighted to find that the biodiversity of *Mortierella*, which was assigned to drought-enriched taxa, had a significant positive correlation with root diameter (*R*^2^ = 0.2532, *P* = 0.02714; Fig. 4e), whereas the biodiversity of *Epicoccum*, which was assigned to drought-depleted taxa, had a significant negative correlation with root diameter (*R*^2^ = 0.2013, *P* = 0.04672; Fig. 4e). These results indicated that *Mortierella* may play a vital role in maintaining plant growth under drought stress by exerting influences on root morphology.

### The drought-enriched fungal strain *M. alpina* enhanced plant drought resistance

**Fig. 5:**
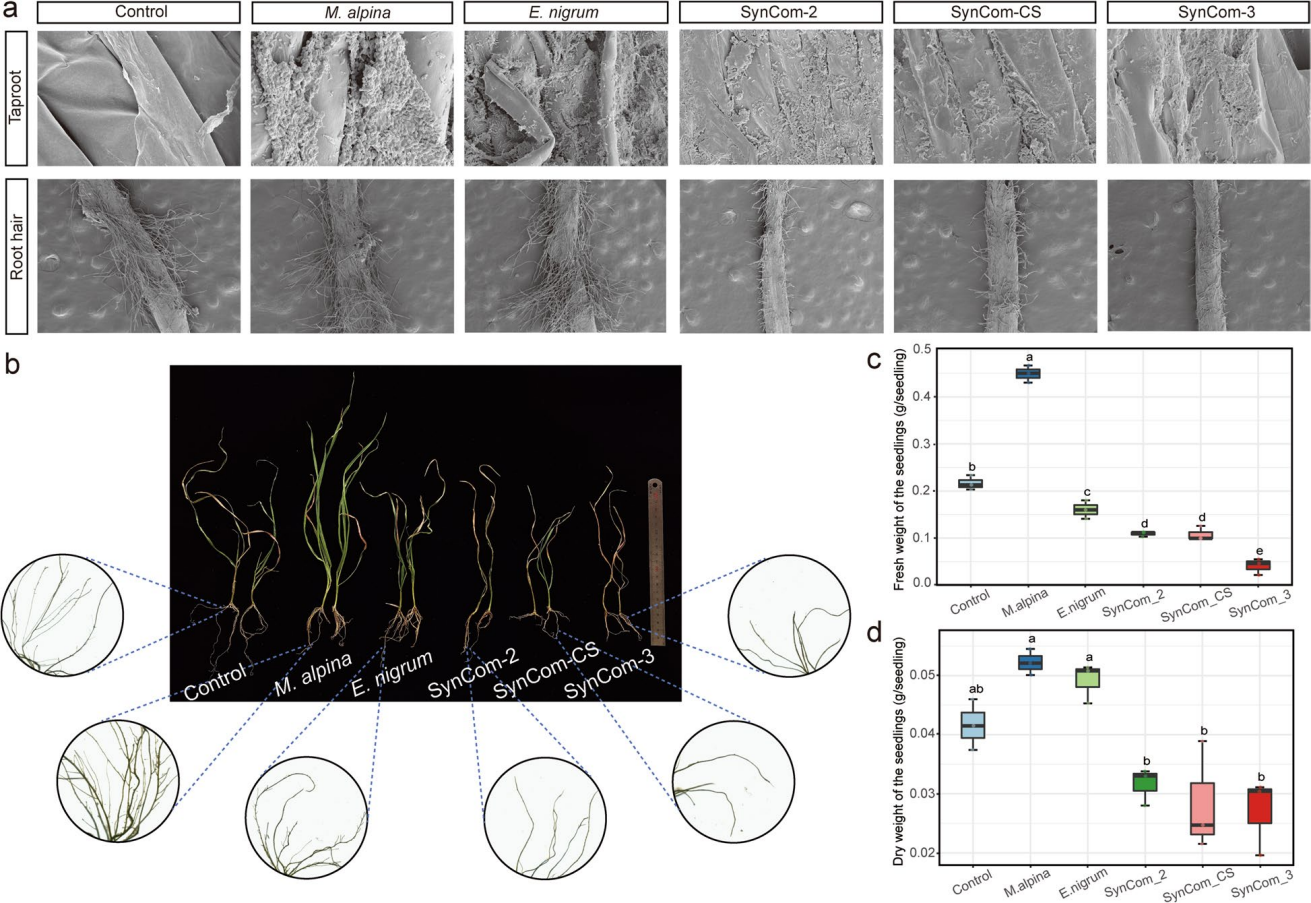
Colonization and drought resistance traits after inoculation of roots of wheat plants. **a** Top: Successful colonization by *M. alpina*, *E. nigrum*, and SynComs under drought stress (10% PEG 6000) of roots of CS wheat. Plants not inoculated with fungi served as controls (scale bars: 10 μm). SynCom-2 consisted of *M. alpina* and *E. nigrum* (scale bars: 20 μm). SynCom-3 comprised *M. alpina*, *E. nigrum*, and SynCom-CS (scale bars: 20 μm). Six biological replicates were measured. Bottom: The effects of SynComs on wheat root tips were recorded using confocal fluorescence microscopy (scale bars: 1 μm). **b** Growth profiles and root phenotypes of wheat in control group and 18 days after inoculation with SynComs under drought stress. Shoot fresh weights (**c**) and dry weights (**d**) of wheat plants in control group and 18 days after inoculation with SynComs under drought stress. A two-sided *t*-test was used for statistical analyses (*n* = 3). *P-value < 0.05, **P-value < 0.01

To confirm the species affiliated to *Mortierella* to enhance plant drought tolerance, we firstly obtained *Mortierella alpina* strains from fungal culture collections and then cultivated the drought-sensitive variety (CS) in Experiment 2 with six different inoculation treatments, namely, (i) controls, (ii) *M. alpina*, (iii) *E. nigrum*, (iv) SynCom-2, (v) SynCom-CS, and (vi) SynCom-3. Our scanning electron microscopy results revealed that both the single fungal strains and the synthetic communities had successfully colonized the surfaces of roots (Fig. 5a). However, we found that the colonized areas after inoculation with a single fungal strain were significantly larger than those after treatment with a synthetic community (first row in Fig. 5a). In comparison with the control group, both *M. alpina* and *E. nigrum* had no significant influence on the development of root hairs. Interestingly, root hair development had been inhibited by inoculation with SynCom-2, SynCom-CS and SynCom-3 (second row in Fig. 5a). In addition, co-colonization by *M. alpina* and *E. nigrum* led to significant inhibitory effects on lateral root growth, whereas these inhibitory effects were absent after inoculation with *M. alpina* or *E. nigrum* alone (Fig. 5b; Table S13). More importantly, we found that CS wheat plants were healthier after inoculation with *M. alpina* (Fig. 5b) and that the fresh weight, as well as the dry weight, of the seedlings increased in comparison with the other five treatments (Fig. 5c, d). Specifically, co-colonization by *M. alpina* and *E. nigrum* significantly impaired plant growth and led to plant death. Similar patterns were observed when plants were inoculated with SynCom-CS and SynCom-3. Taken together, these results clearly indicated that *M. alpina* enriched in the drought-resistant cultivar had the ability to increase the drought tolerance of the drought-sensitive cultivar.

### *M. alpina* activated stress response signaling pathways in the drought-sensitive variety

**Fig. 6:**
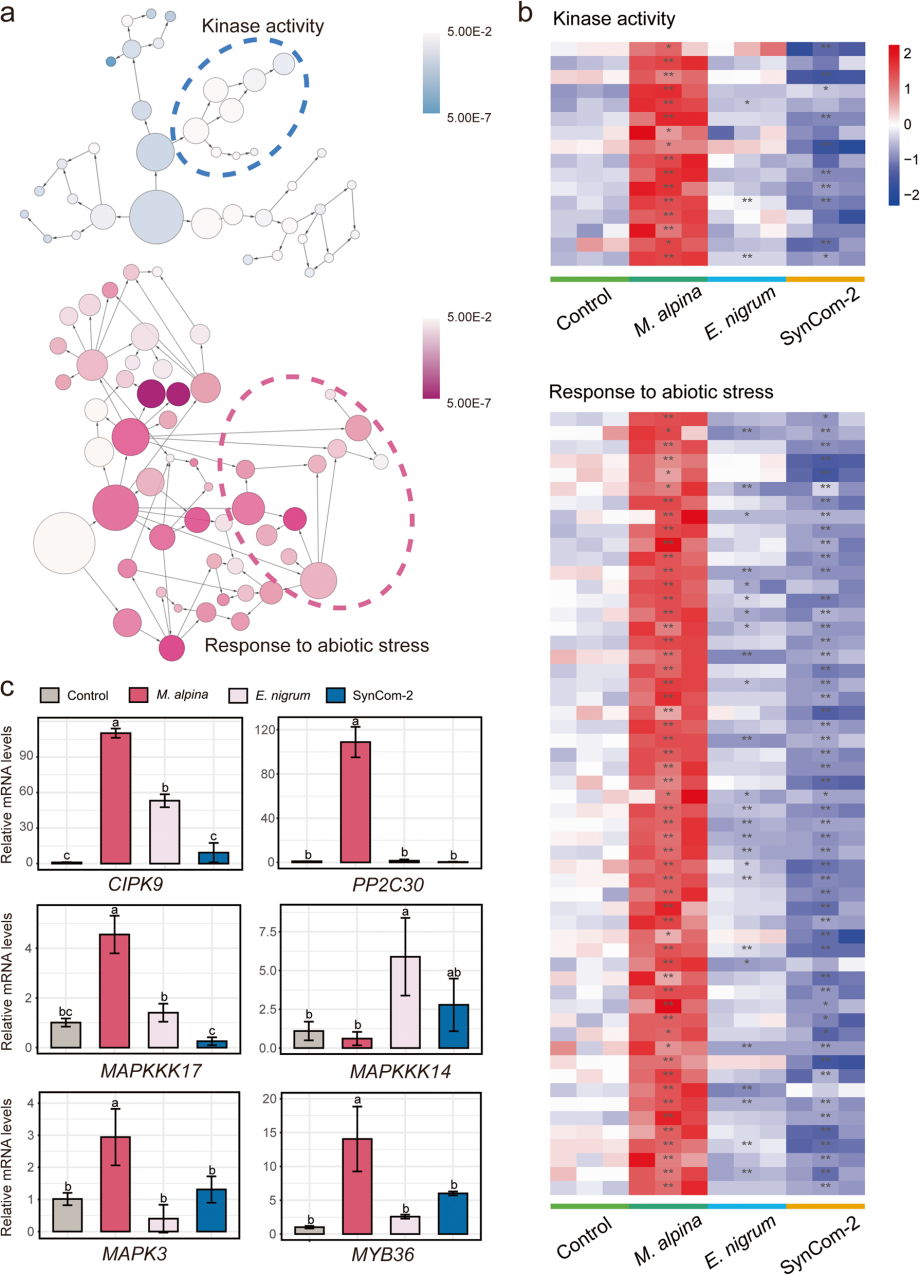
Synergistic responses between *M. alpina* and the host resulted in an increase in drought resistance in wheat. **a** Gene Ontology analysis of differentially expressed genes (DEGs) in cluster 6 in order to identify enriched biological processes, which highlighted a group of abiotic stress response-related and kinase activity that are well known to promote plant responses to drought stress. **b** Heatmaps constructed using gene expression values (normalized log_2_ fold change values) obtained from RNA-Seq data. The gene IDs of DEGs involved in kinase activity and responses to abiotic stress are shown in Table S14. The asterisk displays the difference reached statistical significance (*p < 0.05, **p < 0.01) in *M. alpina*, *E. nigrum*, or SynCom-2 in contrast to control. **c** Relative expression levels (2^−ΔΔCT^) obtained by quantitative real-time PCR analysis

The transcriptome of wheat roots in the absence or presence of *M. alpina* and *E. nigrum* was analyzed in order to get a deeper understanding of the synergistic relation between the plant and fungi in response to drought stress. In this study, a total of 19,216, 11,730, and 14,457 DEGs (treated/control fold change > 1, FDR < 0.05) were obtained for control group vs. *M. alpine* group, control group vs. *E. nigrum group*, and control group vs. Syncom-2 group, respectively (Fig. S15). These DEGs were divided into eight clusters by *k*-means clustering, and these clusters were enriched in specific stress response-related biological functions on the basis of Gene Ontology analysis (Fig. 6a; Fig. S16; Table S14). In accordance with the finding that *M. alpina* enhanced drought tolerance in the drought-sensitive variety (Fig. 5), inoculation with *M. alpina* significantly promoted the expression of specific genes, including those involved in responses to abiotic stress and kinase activity (Fig. 6a, b; Table S15).

We further used qRT-PCR to determine the gene expression levels of 15 DEGs that were detected by RNA-Seq (Fig. 6c; Fig. S17). The induced drought response genes including calcineurin B-like-interacting protein kinase gene (*CIPK9*) and protein phosphatase 2C gene (*PP2C30*) were significantly enriched after treatment with the strain *M. alpina*, with 110 and 108 times higher mRNA levels, respectively, than those in the control group. Synergistic induction by CIPKs and PP2C resistance integrin results in activation of the mitogen-activated protein kinase (MAPK) cascade, including the transcription factors *MYB36*, *MYB62*, and *NAC71*. We also found that inoculation with the strain *M. alpina* resulted in overexpression of *MAPKKK17*, *MAPK3*, and *MYB36* in the drought-sensitive variety under drought stress. In contrast, inoculation with the strain *E. nigrum* significantly increased the mRNA expression of the *MAPKKK14* gene by a factor of around 6.2 in comparison with inoculation with the strain *M. alpina*. Therefore, the strain *M. alpina* enhanced the drought resistance of plants, which can be attributed to synergistic regulation of abiotic response-related pathways, in addition to overexpression of MAPK signaling cascades, which is known to contribute to fungi-enhanced drought tolerance in plants.

Furthermore, the mRNA expression levels of the genes *MYB36* and *WOX11* associated with lateral root development increased significantly and were more than 20 and 2 times higher, respectively, than those in the control group (*p* < 0.05) in CS wheat after inoculation with the strains *M. alpina* or *E. nigrum*. However, the mRNA expression levels of *MYB36* exhibited no significant difference and those of *WOX11* decreased significantly in CS wheat after inoculation with SynCom-2 (Fig. S18). These results revealed that not only had SynCom-2 inhibited the abovementioned gene expression, but also had significant inhibitory effects on the root length, number of root nodes, and number of root tips compared with control group (Fig. S19).

## Discussion

In this study, we sought to verify whether the harnessed rhizosphere microbiome from the drought-resistant wheat cultivar have the ability to enhance the drought tolerance of drought-sensitive wheat under drought stresses using amplicons, metagenomics, and RNA-sequencing approaches. By profiling both bacterial and fungal communities in the drought-resistant and drought-sensitive cultivars at two different planting sites, we reveal that wheat genotypes exert significantly influences on the rhizosphere microbiomes, and drought-resistant wheat cultivar has more diverse bacterial and fungi taxa than drought-sensitive cultivar. Microbial interkingdom association network analysis indicates that the drought-resistant wheats possess higher network complexity of rhizosphere microbiomes, regardless of planting sites. Metagenomics sequencing data from these two wheat cultivars further suggest that the enriched rhizosphere microbiome affiliate to drought-resistant cultivar has greater investment in “Energy metabolism” and “Carbohydrate metabolism”. Moreover, our work provide evidence that colonization of the single strain *M. alpine* significantly enhances the plant growth under drought stresses. Through RNA-Seq, we further confirm that the strains *M. alpine* heighten plant adaptability of drought-sensitive cultivar to drought stresses through synergistic regulation of CIPK and PP2C genes. Below, we discuss how these findings have facilitated our understating of the single fungi strains confer drought tolerance to plant hosts.

### Host drought-resistant differentiation shapes distinct rhizosphere microbiome assembly and functional adaptation

Host plants and their associated microbes interacted by multiple distinct mechanisms, including plant-to-microbiome, microbiome-to-microbiome, and microbiome-to-host, which sustains agricultural ecosystem services in response to ongoing environmental changes [28, 52, 64]. Our results demonstrated that the drought-resistant wheat cultivar possessed more diverse bacterial and fungal communities, particularly for specific fungal species. The consequences of ecological memory of recurrent drought to the soil microbial community [7] and alternations in the root exudates of plants under the non- continuous drought stresses [58, 74] could at least partially explain this altered assembly of specific microbial taxa. Furthermore, multiple microbial attributes of the rhizosphere microbiome were jointly influenced by the plant genotype and planting site. These findings are consistent with previous studies [32, 66, 71], suggesting that host plant signature drives the differentiation in the ecological role of rhizosphere microbiomes in agricultural ecosystems. Increasing crop yields and adaptation of plants to drought stress are among the most important goals during the breeding process of drought-resistant wheats. Rhizosphere and root-associated microbiota play an essential role in plant growth and resilience [59]. The impact of plant breeding on the rhizosphere microbiome assemblage and functional profiles usually represents directional selection by root exudates that are released by plants with different genotypes [44, 58]. Moreover, wheat varieties with different drought tolerances could recruit specific microbiota that formed complex microbial interkingdom association networks to reinforce the resistance of plants under harsh environmental conditions. We further found that the drought-resistant wheat cultivar possessed more complex bacterial–fungal interkingdom correlations with a higher proportion of negative edges in the microbial networks. Pioneer studies indicated that complex networks with greater connectivity are more resilient to environmental perturbations than networks with lower connectivity [56, 76]. It has also been shown that the complexity of microbial networks contributed greatly to ecosystem multifunctionality [65]. In this sense, the higher complexity and proportion of negative edges between hub bacteria and fungi may indicate that rhizosphere microbiota derived from the drought-resistant wheat cultivar are more tolerant of drought stress, as different bacteria and fungi can complement each other. Interestingly, we found that plant pathogen fungi exhibited significantly higher abundance in the warmer climate area (e.g., SQ site) than semi-arid area (e.g., YL site), suggesting that warming climates may facilitate the outbreak of plant pathogenic fungi and further affects the plant growth via pathogenic-to-beneficial microbe interactions [17, 54].

Apart from changing rhizosphere microbiome assemblages and interkingdom association networks, plant–microbe co-adaptation plays key roles in shaping functional adaptations of rhizosphere microbiomes in response to stresses [15]. Metagenomic analyses in our study revealed that the functional diversity and enriched functional profiles in rhizosphere microbiomes from the drought-resistant cultivar exhibited contrasting patterns in comparison with the drought-sensitive cultivar. Rhizosphere microbiomes belonging to the drought-resistant cultivar had greater relative abundances of genes involved in “Carbohydrate metabolism” and “Energy metabolism”, whereas functional genes encoding factors associated with cell integrity and involved in “Membrane transporter” and “Cell growth and death” were significantly enriched in the drought-sensitive cultivar. This is likely due to the rhizosphere microbiome from drought-resistant cultivar and drought-sensitive cultivar adopt distinct life history strategies to confront drought stress in the agroecosystem [45]. In the framework of trait-based life history theories in microbial ecology, drought-resistant microbes may adopt growth yield and stress tolerance strategies to cope with harsh conditions. On the other hand, drought-sensitive microbes tend to adopt resource acquisition strategy to maintain the microbial cellular osmolarity and integrity under adverse conditions. Tradeoffs in microbial life history can have consequences for turnover and transformation of soil availability nutrients [11], thereby directly mediating the gene expression of plants via microbiome-to-host interactions [20]. More importantly, rhizosphere microbiomes from drought-resistant cultivar with enriched “Energy metabolism”, in particular in “Carbon fixation” and “Nitrogen metabolism”, may reinforce the ability and flexibility of crops to deal with diverse environmental stresses by enhancing the acquisition of carbon and nitrogen for plants [16]. These results indicate that plant hosts exert a strong selective effect on the utilization of specific metabolic functions of rhizosphere microbiota. Complementary to the findings of pioneer studies that selection by the host plant via genetic features plays a key role in shaping rhizosphere microbiome communities [60, 61], this work provides novel evidence that host plant signature profoundly influence not only ecological patterns of rhizosphere microbiomes but also their functional adaptations. Collectively, these findings offer a gateway to the manipulation of specific functions of bacteria and fungi to enhance the drought resistance of wheats during the plant breeding process.

### SynComs strongly inhibit the development of lateral roots and root hairs in wheat

Plant root traits are central drivers of many ecosystem processes, plant roots can quickly respond to harsh environments in natural and agricultural ecosystems [69]. More importantly, root morphological features are vital indicators of plant hosts in responses to drought stress [43]. A pioneer study also highlighted the critical role of dominant microbiota in shaping the phenotypic traits of plant roots when confronting drought stress [42]. Importantly, long and thick L-type of lateral roots is better suited for water uptake during drought stress [19]. Plants exhibit substantial variation in root morphology in response to drought stresses and resource availability, but our understanding of how specific rhizosphere microbiomes covary with root morphology remains inconclusive.

Here, we found that lateral roots and root hairs of the drought-susceptible cultivar were strongly inhibited by co-colonization by *M. alpina* and *E. nigrum* under drought stress. The underlying mechanisms currently include (i) competition for niche space, which directly determined whether the beneficial rhizosphere microbiome successful colonization on the root surface [9, 14, 25],(ii) inhibition from secondary metabolites, which determined metabolite exchange networks between rhizosphere microbiome and plant roots via potential allelopathy effect [41, 58],(iii) regulation of gene expression in plants, which would affected plant hormone signal transduction and immune system activities (tolerance or avoidance) via the process of microbial inheritance in plants [1, 38]. Furthermore, pioneer studies indicated that root hairs are able to influence the rhizosphere microbiome assemblage, and in turn rhizosphere microbes are able to interact with plant-modifying root hairs [34, 55]. Root hairs have physical properties that play vital roles in the two-way interaction by specifically changing the plant–microbe interaction interface [44]. MYB36 is known to be a novel factor involved in the later stages of lateral root development and is expressed in cells surrounding lateral root primordia to regulate the proliferation–differentiation transition in the root meristem [18]. WOX11 is expressed in the founder cells of adventitious roots and activates LBD16, which has vital functions in the formation of lateral roots and adventitious roots [77]. SynCom-2 significantly inhibited the development of lateral roots and root hairs by significantly decreasing the transcription levels of *MYB36* and *WOX11*, which suggested that water uptake and nutrient acquisition for the plant had been impeded, which led to plant death. Although the fungal strain *M. alpina* positively contributed to the resistance of wheat to drought, it is worth noting that the fungal strain *E. nigrum* may have extraordinarily eliminated the positive role of *M. alpina* when these two fungi co-colonized the plant rhizosphere. It was suggested that a single fungal strain enriched from the drought-resistant variety maintained root growth. In contrast, the synthetic microbial community contained fungal strains from the drought-sensitive cultivar, which could have hampered plant growth by inhibiting lateral root development. Our study provides empirical evidence that fungal communities utilized by drought-resistant wheat may play an increasing ecological role in sustaining plant growth and root development. These findings provide new insights into the complex interactions between root morphological traits and rhizosphere microbiomes and pave the way to the deployment of “beneficial microorganisms” for sustainable agriculture. However, the dynamic interactions among root exudates, root morphology, and rhizosphere microbiomes are not fully understood and need further exploration.

### *M. alpina* enhances the drought tolerance of wheat by activating the CIPK-PP2C network

There is mounting evidence that drought leads to dramatic shifts in rhizosphere microbiomes and in which rhizosphere microbiomes are selected by plant hosts [16]. However, the feedback effects of drought-resistant microbiomes on plant growth and fitness are still limited. Drought-resistant wheat recruits the genotype-specific fungus *Mortierella*, which is directly responsible for increasing the efficiency of the uptake of nutrients, including P and Fe, and the synthesis of triacylglycerols, phytohormones (e.g., indole-3-acetic acid), and 1-aminocyclopropane-1-carboxylate deaminase [49]. Apart from direct mechanism, *Mortierella* also strengthen indirect mechanism activities, including hydrogen cyanide, chitinase, protease, and antibiotic for plant growth promotion [27, 53], which results in a positive effect on the protection of crops against harsh conditions [8, 36]. In this study, *M. alpina* acted as a beneficial fungus with the ability to enhance the drought resistance of the drought-susceptible cultivar. When the host suffered from drought stress, *M. alpina* and the host generated synergistic responses by activating the CIPK-PP2C network. In accordance with a pioneer study, CIPK-PP2C complexes could act as molecular on–off switches to regulate the phosphorylation state of plant ion transporters involved in stress responses [35]. In particular, transcriptional regulation of *CIPK9* and *PP2C30* resulted in 100 times higher basal expression levels in wheat inoculated with *M. alpina* in comparison with the control group because of the strong synergistic effects of MAPKs. A previous study indicated that multiple MAPKs can be quickly activated in plants to cope with drought stress [78]. Our qRT-PCR data further confirmed that *M. alpina* regulated the gene expression of *MAPKKK17* and *MAPK3* in the drought-susceptible variety in response to abiotic stress. This result indicated that multiple stress signaling pathways, which have crosstalk features, were directly or indirectly activated to form complex signal transduction networks in host plants by colonization by *M. alpine* (Fig. S18). On the basis of these findings, we propose a scheme where beneficial fungi play a key role in strengthening the drought resistance of plants by activating plant integrated responses to abiotic stimuli, although the molecular triggers of these synergistic responses remain to be characterized.

## Conclusions

**Fig. 7:**
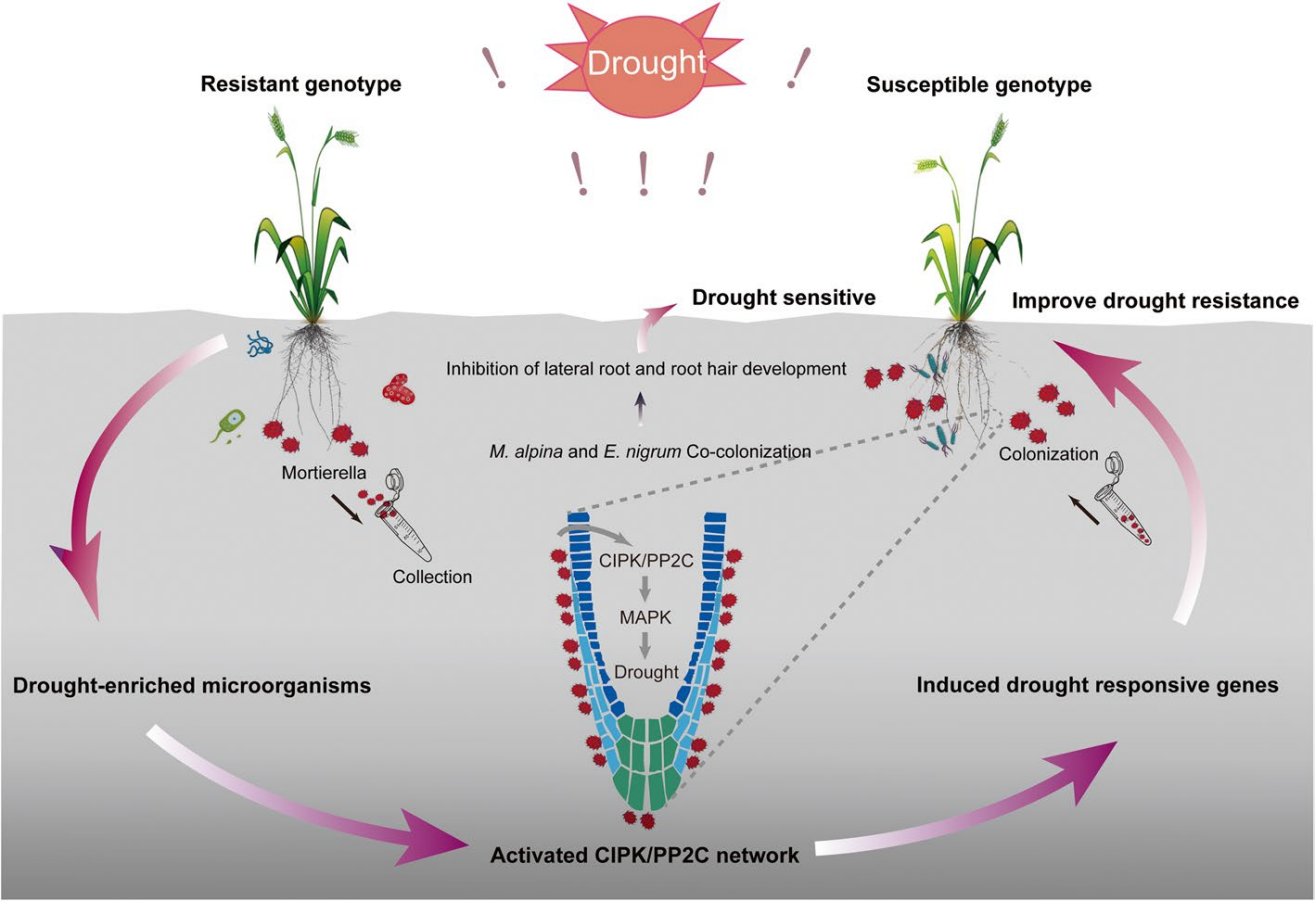
Conceptual paradigm depicting the synergistic mechanisms between host plants and rhizosphere microorganisms that improve drought resistance in wheat. The drought-resistant wheat cultivar determines the direct response of plants to recruit genotype-specific microbial communities, such as *M. alpina*. *M. alpina* can feedback to plants and improve drought resistance by activating the CIPK-PP2C network to induce drought-responsive genes. However, *M. alpina* and *E. nigrum* together have a negative effect on the lateral roots and root hairs of wheat and lead to wheat becoming significantly more sensitive to drought stress

Manipulation of rhizosphere microbiomes represents an effective and promising strategy for addressing the threat that changes in global climate such as drought pose to sustainable agriculture. We propose a conceptual paradigm that demonstrates that the genotype of the drought-resistant wheat cultivar restructures the rhizosphere microbiome and shapes functional adaptations and highlights the importance of beneficial fungal strains in enhancing the drought resistance of wheat (Fig. 7). More importantly, the rhizosphere microbiomes that were recruited by the drought-resistant genotype could serve as a “microorganism goldmine” for the design of reasonable synthetic communities for increasing agricultural productivity. Taken together, our results represent a significant advance in determining the molecular mechanisms of drought-enriched fungal strains in enhancing the drought resistance of plants and provide critical new knowledge on key ecological interactions between drought-resistant wheats and their rhizosphere microbiomes. These suggest that a functionally reliable “beneficial biome” will offer opportunities for sustainable agriculture and provide a new direction for breeding drought-resistant wheat and enhancing agricultural sustainability under global climate change scenarios.

### Supplementary Information

**Additional file 1: Table S1.** Annual precipitation and average monthly precipitation at the Suqian (SQ) and Yangling (YL) planting sites. **Table S2.** Primers used for quantitative real-time PCR (qRT-PCR) in this study. **Table S3.** Effects of genotypes, planting sites, and niche compartments on the bacterial and fungal communities based on PERMANOVA analysis. **Table S4.** Relative abundances of dominant bacterial phyla for YH and CS wheats at the SQ and YL sites. **Table S5.** Relative abundances of dominant fungal phyla for YH and CS wheats at SQ and YL sites. **Table S6.** Relative abundances of drought-responsive bacterial OTUs at genus level. **Table S7.** Relative abundances of drought-responsive fungal OTUs at genus level. **Table S8.** The absolute abundance of plant pathogenic OTUs at phyla and genus level. **Table S9.** Topological characteristics of the microbial interkingdom association networks in the YH and CS sites. **Table S10.** Metagenomics sequencing data characteristics for rhizosphere microbiomes from different wheat cultivars at the SQ and YL sites. **Table S11.** Root morphology traits of wheat sample under different microbial inoculation treatments in the Experiment 1. **Table S12.** Spearman’s Correlation coefficients between rhizosphere microorganisms and root morphological traits. **Table S13.** Root morphology traits of China Spring (CS) in the Experiment 2. **Table S14.** Division by k-means clustering of differentially expressed genes (DEGs) into clusters 8. **Table S15.** Gene IDs of DEGs involved in responses to abiotic stress and mitogen-activated protein Kinase activity. **Fig. S1.** Rhizosphere soil microbiome diversity and distribution patterns. (a) Canonical analysis of principal coordinates (CAP) of bacteria and fungi was performed to determine whether there were differences in samples according to the Bray–Curtis dissimilarity matrix. (b) Distinct beta-diversity between bacterial and fungal communities was shown by principal coordinate analysis ordinations (PCoA). Abbreviations: Suqian site (SQ), Yangling site (YL), drought-susceptible wheat cultivar Chinese Spring (CS), drought-resistant wheat cultivar Yunhan 618 (YH), bulk soil (B), and rhizosphere soil (T). **Fig. S2.** Soil microbiome diversity in bulk and rhizosphere soils. (a) Results of CAP of bacterial and fungal microbiota in bulk and rhizosphere soils. (b) PCoA plots of the bacterial and fungal community structure. **Fig. S3.** Phylogenetic tree for bacterial (a) and fungal (b) communities at the phylum level showing the hierarchical relationships of operational taxonomic units (OTUs) with an average abundance of greater than 3000 in each sample population (inner circle). The outer rings indicate the abundance and distribution of OTUs in all samples. The values indicate the average abundance of each OTU (log-transformed). The colors represent the OTUs at the phylum level. **Fig. S4.** Differential abundance of bacteria and random forest classification revealing differences in rhizosphere microbial structure. (a) and (b) Volcano plots visualizing the enrichment and depletion patterns of bacteria in YH in comparison with CS at SQ (a) and YL (b). Black dots represent no significant differences in OTUs. Red and blue dots indicate an individual OTU significantly enriched in YH and CS, respectively. (c) and (d) The influence of YH and CS at SQ (c) and YL (d) on the 11 most important bacterial taxa in rhizosphere soil was determined by random forest classification. The relative abundance of rhizobacteria differed in accordance with the drought tolerance of the sample. OTUs are arranged along the y-axis in descending order of importance by calculating the Gini coefficient to validate the accuracy of the model. For the abbreviations see Fig. S1. **Fig. S5.** Differential abundance of fungi and random forest classification revealing differences in rhizosphere microbial structure. (a) and (b) Volcano plots visualizing the enrichment and depletion patterns of fungi in YH in comparison with CS at SQ (a) and YL (b). (c) and (d) The influence of YH and CS at SQ (c) and YL (d) on the five most important fungal taxa in rhizosphere soil was determined by random forest classification. **Fig. S6.** Heatmap showing the relative abundances of abundant genera present in drought-responsive bacteria and fungi, which varied between the drought-resistant cultivar and the drought-susceptible cultivar at the two sites. All taxa are indicated according to their significance (Wilcox test, *P* < 0.05) in the different samples. The colors represent the log_2_ fold change (log_2_FC) in relative abundance. **Fig. S7.** The pathogenic ASVs included in the fungi phyla Basidiomycota, Blastocladiomycota, and Chytridiomycota for all samples. **Fig. S8.** PCoA plots of functional traits of the rhizosphere bacterial and fungal communities. (a) PCoA plots of functional traits using relative abundances based on Kyoto Encyclopedia of Genes and Genomes (KEGG) pathway level 3, (b) orthologous groups of proteins (COG), and (c) carbohydrate-active enzymes (CAZyome). **Fig. S9.** Diversity indices for functional categories, including KEGG pathway level 3 (a), CAZyome (b), and COG (c), for each sample from rhizosphere soil. A difference in the letters on top of each boxplot suggests a statistically significant difference according to one-way analysis of variance (*P* < 0.05). **Fig. S10.** Volcano plots visualizing the KEGG Orthology (KO) pathways and functional categories. (a) and (b) Volcano plots visualizing the KO pathways that were significantly enriched or depleted in the drought-resistant cultivar in comparison with the drought-susceptible cultivar at the YL and SQ sites. DI and DSI represent the depletion index and dissimilarity index, respectively. Grey dots indicate that there were no significant differences in OTUs. Dark blue and yellow dots indicate an individual OTU significantly enriched in the drought-resistant cultivar and the drought-susceptible cultivar, respectively. (c) and (d) Volcano plots visualizing the KO functional categories that were significantly enriched or depleted in the drought-resistant cultivar in comparison with the drought-susceptible cultivar at the YL and SQ sites. **Fig. S11.** Functional profile based on KEGG pathway level 3 (a), CAZyome (b), and COG (c) of top OTUs in four samples. **Fig. S12.** Functional profiles of rhizosphere microbiomes based on KO. KO functional categories and pathways that were significantly enriched in the drought-susceptible cultivar and drought-resistant cultivar at the YL (a) and SQ (b) sites. Red and dark blue colors represent significantly enriched KO pathways in YH and CS wheats, respectively. **Fig. S13.** Relative abundance of functional genes associated with drought responses that varied between the drought-resistant cultivar and the drought-susceptible cultivar. **Fig. S14.** Variations in rhizosphere microbial communities explaining differences in wheat root system architecture. Keystone bacterial and fungal OTUs significantly correlated with root system architecture in wheat grown in Hoagland nutrient solution treated with microbial suspensions (log_2_FC > 2, *p* < 0.05). Heatmap displaying the top 30 bacterial OTUs and 30 fungal OTUs on the basis of relative abundance distributions. Blue in the top panel indicates that the OTU was enriched in CS, and orange indicates that the OTU was enriched in YH. *p < 0.05, **p < 0.01, and ***p < 0.001. **Fig. S15.** DEGs in *M. alpina*, *E. nigrum*, and SynCom-2 and results of KO functional analyses. Volcano plots of DEGs in wheats grown in Hoagland nutrient solution with or without fungal inoculation under drought stress. The threshold for statistically significant differential expression was fold change > 1 and q-value < 0.05. Upregulated genes are shown in red, and downregulated genes are shown in blue. **Fig. S16.** Division of all DEGs into eight clusters by k-means clustering analysis. **Fig. S17.** Relative expression levels (2^−ΔΔCT^) of drought response-related genes obtained by qRT-PCR analysis. **Fig. S18.** Relative expression levels (2^−ΔΔCT^) of root development-related genes obtained by RNA-seq analysis. **Fig. S19.** Root-related parameters of China Spring (CS) in the Experiment 2. **Fig. S20.** Model of *M. alpina*-induced stress response signaling in wheat. *M. alpina* strongly induces expression of genes in the CIPK and PP2C families, CIPK-PP2C complexes activate *MAPKKK17*, and the activated MAPK cascade induces the expression of the transcription factors MYB36, MYB62, and NAC71 to regulate the expression of drought-responsive genes. However, suppression by* E. nigrum* of expression of the *MAPK6* and *NAC71* genes leads to repression of drought-responsive genes.

**Additional file 2.**

**Additional file 3.**

**Additional file 4.**

**Additional file 5.**

**Additional file 6.**

**Additional file 7.**

## Data Availability

The datasets generated for the current study are available for the public. All data including bacteria and fungi from amplicon sequencing was deposited at The National Center for Biotechnology Information (NCBI) with the accession numbers PRJNA789431. For the shotgun metagenomics sequencing and RNA sequencing, the accession numbers of raw data are PRJNA1013685 and PRJNA904648, respectively. The core codes with R script that support the findings of this study have been deposited in the github platform (https://github.com/Duntao/Project_of_Drought_Microbiome). The rarefied operational taxonomic unit table of bacteria and fungi in field experiment includes taxonomic information presented in Dataset S1 and Dataset S2, respectively. The microbial functional information from metagenomics sequencing includes KEGG, NOG, and CAZy profiles presented in Dataset S3, Dataset S4, and Dataset S5, respectively. The gene expression matrix from the RNA sequencing in the greenhouse experiment are shown in Dataset S6.
