# Correction: Host genotype-specific rhizosphere fungus enhances drought resistance in wheat

**DOI:** 10.1186/s40168-024-01794-0

**Published:** 2024-03-23

**Authors:** Hong Yue, Xuming Sun, Tingting Wang, Ali Zhang, Dejun Han, Gehong Wei, Weining Song, Duntao Shu

**Affiliations:** 1 College of Agronomy, National Key Laboratory of Crop Improvement for Stress Tolerance and Production, Northwest A&F University, Yangling, 712100 Shaanxi China; 2 College of Life Sciences, National Key Laboratory of Crop Improvement for Stress Tolerance and Production, Northwest A&F University, Yangling, 712100 Shaanxi China; 3 Shaanxi Key Laboratory of Agricultural and Environmental Microbiology, Yangling, 712100 Shaanxi China

**Correction: Microbiome 12, 44 (2024)**

**https://doi.org/10.1186/s40168-024-01770-8**

Following publication of the original article [1], the author reported that in Figs. 4D and 4E the heatmap were missing.

The incorrect Fig. 4 is
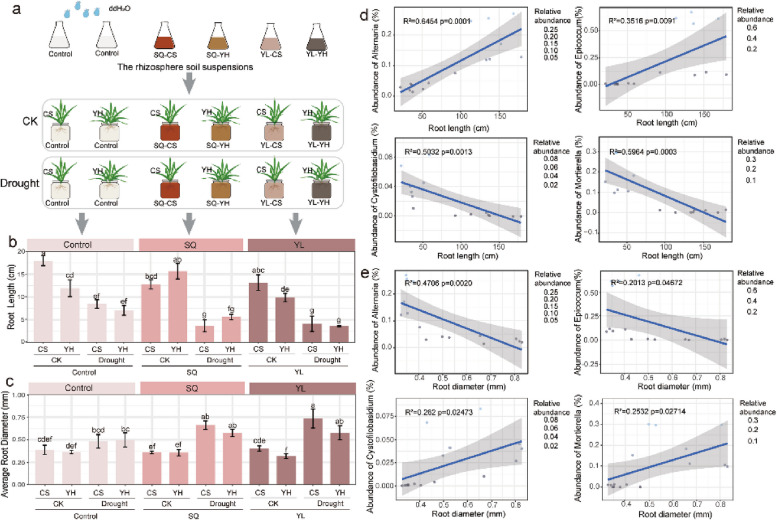


The correct Fig. 4 is
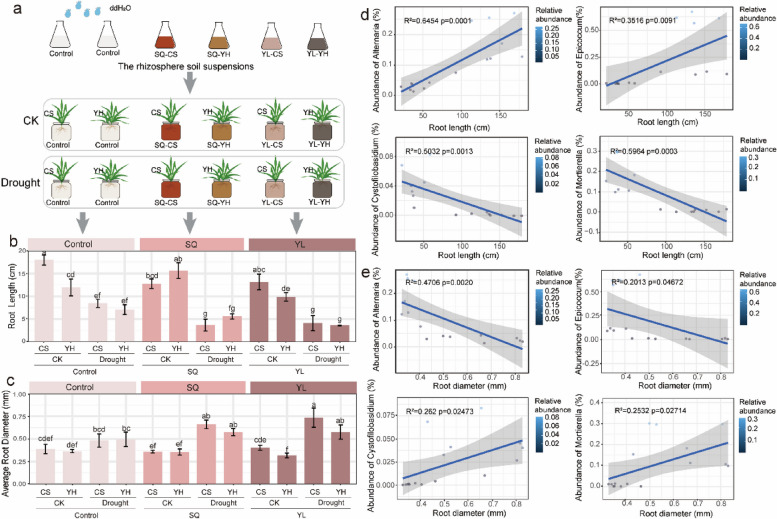


The original article has been updated.
