# Supplementary material for: Host genotype-specific rhizosphere fungus enhances drought resistance in wheat
Source: Microbiome. 2024 Mar 4;12:44. doi: 10.1186/s40168-024-01770-8 (PMC10910722; doi:10.1186/s40168-024-01770-8)

**Supporting Information**

**Host genotype-specific rhizosphere fungus enhances drought resistance in wheat**

Hong Yue^1#^, Xuming Sun^1#^, Tingting Wang^1^, Ali Zhang^1^, Dejun Han^1^,

Gehong Wei^2, 3 ***^, Weining Song^1 **^, Duntao Shu^2, 3 *^

^1^ College of Agronomy, National Key Laboratory of Crop Improvement for Stress Tolerance and Production, Northwest A&F University, Yangling, Shaanxi 712100, China

^2^ College of Life Sciences, National Key Laboratory of Crop Improvement for Stress Tolerance and Production, Northwest A&F University, Yangling, Shaanxi 712100, China

^3^ Shaanxi Key Laboratory of Agricultural and Environmental Microbiology, Yangling, Shaanxi 712100, China

^#^ These authors contributed equally to this work

^*^ Corresponding author: [donald.shu@nwafu.edu.cn](mailto:donald.shu@nwafu.edu.cn) (D. Shu)

^**^ Corresponding author: sweining2002@yahoo.com (W. Song)

^***^ Corresponding author: [weigehong@nwafu.edu.cn](mailto:weigehong@nwafu.edu.cn) (G. Wei)

Tel: 86-18710994335; Fax: 86-29-87090162

**Contents**

**1. Tables**

**Table S1** Annual precipitation and average monthly precipitation at the Suqian (SQ) and Yangling (YL) planting sites.

**Table S2** Primers used for quantitative real-time PCR (qRT-PCR) in this study.

**Table S3** Effects of genotypes, planting sites, and niche compartments on the bacterial and fungal communities based on PERMANOVA analysis.

**Table S4** Relative abundances of dominant bacterial phyla for YH and CS wheats at the SQ and YL sites.

**Table S5** Relative abundances of dominant fungal phyla for YH and CS wheats at SQ and YL sites.

**Table S6** Relative abundances of drought-responsive bacterial OTUs at genus level.

**Table S7** Relative abundances of drought-responsive fungal OTUs at genus level.

**Table S8** The absolute abundance of plant pathogenic OTUs at phyla and genus level.

**Table S9** Topological characteristics of the microbial interkingdom association networks in the YH and CS sites.

**Table S10** Metagenomics sequencing data characteristics for rhizosphere microbiomes from different wheat cultivars at the SQ and YL sites.

**Table S11** Root morphology traits of wheat sample under different microbial inoculation treatments in the Experiment 1.

**Table S12** Spearman’s Correlation coefficients between rhizosphere microorganisms and root morphological traits.

**Table S13** Root morphology traits of China Spring (CS) in the Experiment 2

**Table S14** Division by k-means clustering of differentially expressed genes (DEGs) into clusters 8.

**Table S15** Gene IDs of DEGs involved in responses to abiotic stress and mitogen-activated protein Kinase activity.

**2. Figures**

**Fig. S1** Rhizosphere soil microbiome diversity and distribution patterns. **(a)** Canonical analysis of principal coordinates (CAP) of bacteria and fungi was performed to determine whether there were differences in samples according to the Bray–Curtis dissimilarity matrix. **(b)** Distinct beta-diversity between bacterial and fungal communities was shown by principal coordinate analysis ordinations (PCoA). Abbreviations: Suqian site (SQ), Yangling site (YL), drought-susceptible wheat cultivar Chinese Spring (CS), drought-resistant wheat cultivar Yunhan 618 (YH), bulk soil (B), and rhizosphere soil (T).

**Fig. S2** Soil microbiome diversity in bulk and rhizosphere soils. (**a)** Results of CAP of bacterial and fungal microbiota in bulk and rhizosphere soils. **(b)** PCoA plots of the bacterial and fungal community structure.

**Fig. S3** Phylogenetic tree for bacterial **(a)** and fungal **(b)** communities at the phylum level showing the hierarchical relationships of operational taxonomic units (OTUs) with an average abundance of greater than 3000 in each sample population (inner circle). The outer rings indicate the abundance and distribution of OTUs in all samples. The values indicate the average abundance of each OTU (log-transformed). The colors represent the OTUs at the phylum level.

**Fig. S4** Differential abundance of bacteria and random forest classification revealing differences in rhizosphere microbial structure. **(a)** and **(b)** Volcano plots visualizing the enrichment and depletion patterns of bacteria in YH in comparison with CS at SQ **(a)** and YL **(b)**. Black dots represent no significant differences in OTUs. Red and blue dots indicate an individual OTU significantly enriched in YH and CS, respectively. **(c)** and **(d)** The influence of YH and CS at SQ **(c)** and YL **(d)** on the 11 most important bacterial taxa in rhizosphere soil was determined by random forest classification. The relative abundance of rhizobacteria differed in accordance with the drought tolerance of the sample. OTUs are arranged along the y-axis in descending order of importance by calculating the Gini coefficient to validate the accuracy of the model. For the abbreviations see Fig. S1.

**Fig. S5** Differential abundance of fungi and random forest classification revealing differences in rhizosphere microbial structure. **(a)** and **(b)** Volcano plots visualizing the enrichment and depletion patterns of fungi in YH in comparison with CS at SQ **(a)** and YL **(b)**. **(c)** and **(d)** The influence of YH and CS at SQ **(c)** and YL **(d)** on the five most important fungal taxa in rhizosphere soil was determined by random forest classification.

**Fig. S6** Heatmap showing the relative abundances of abundant genera present in drought-responsive bacteria and fungi, which varied between the drought-resistant cultivar and the drought-susceptible cultivar at the two sites. All taxa are indicated according to their significance (Wilcox test, *P* < 0.05) in the different samples. The colors represent the log_2_ fold change (log_2_FC) in relative abundance.

**Fig. S7** The pathogenic ASVs included in the fungi phyla Basidiomycota, Blastocladiomycota, and Chytridiomycota for all samples.

**Fig. S8** PCoA plots of functional traits of the rhizosphere bacterial and fungal communities. **(a)** PCoA plots of functional traits using relative abundances based on Kyoto Encyclopedia of Genes and Genomes (KEGG) pathway level 3, **(b)** orthologous groups of proteins (COG), and **(c)** carbohydrate-active enzymes (CAZyome).

**Fig. S9** Diversity indices for functional categories, including KEGG pathway level 3 **(a)**, CAZyome **(b)**, and COG **(c)**, for each sample from rhizosphere soil. A difference in the letters on top of each boxplot suggests a statistically significant difference according to one-way analysis of variance (*P* < 0.05).

**Fig. S10** Volcano plots visualizing the KEGG Orthology (KO) pathways and functional categories. **(a)** and **(b)** Volcano plots visualizing the KO pathways that were significantly enriched or depleted in the drought-resistant cultivar in comparison with the drought-susceptible cultivar at the YL and SQ sites. DI and DSI represent the depletion index and dissimilarity index, respectively. Grey dots indicate that there were no significant differences in OTUs. Dark blue and yellow dots indicate an individual OTU significantly enriched in the drought-resistant cultivar and the drought-susceptible cultivar, respectively. **(c)** and **(d)** Volcano plots visualizing the KO functional categories that were significantly enriched or depleted in the drought-resistant cultivar in comparison with the drought-susceptible cultivar at the YL and SQ sites.

**Fig. S11** Functional profile based on KEGG pathway level 3 **(a)**, CAZyome **(b)**, and COG **(c)** of top OTUs in four samples.

**Fig. S12** Functional profiles of rhizosphere microbiomes based on KO. KO functional categories and pathways that were significantly enriched in the drought-susceptible cultivar and drought-resistant cultivar at the YL **(a)** and SQ **(b)** sites. Red and dark blue colors represent significantly enriched KO pathways in YH and CS wheats, respectively.

**Fig. S13** Relative abundance of functional genes associated with drought responses that varied between the drought-resistant cultivar and the drought-susceptible cultivar.

**Fig. S14** Variations in rhizosphere microbial communities explaining differences in wheat root system architecture. Keystone bacterial and fungal OTUs significantly correlated with root system architecture in wheat grown in Hoagland nutrient solution treated with microbial suspensions (log_2_FC > 2, *p* < 0.05). Heatmap displaying the top 30 bacterial OTUs and 30 fungal OTUs on the basis of relative abundance distributions. Blue in the top panel indicates that the OTU was enriched in CS, and orange indicates that the OTU was enriched in YH. **p* < 0.05, ***p* < 0.01, and ****p* < 0.001.

**Fig. S15** DEGs in *M. alpina*, *E. nigrum*, and SynCom-2 and results of KO functional analyses. Volcano plots of DEGs in wheats grown in Hoagland nutrient solution with or without fungal inoculation under drought stress. The threshold for statistically significant differential expression was fold change > 1 and q-value < 0.05. Upregulated genes are shown in red, and downregulated genes are shown in blue.

**Fig. S16** Division of all DEGs into eight clusters by k-means clustering analysis.

**Fig. S17** Relative expression levels (2^−ΔΔCT^) of drought response-related genes obtained by qRT-PCR analysis.

**Fig. S18** Relative expression levels (2^−ΔΔCT^) of root development-related genes obtained by RNA-seq analysis.

**Fig. S19** Root-related parameters of China Spring (CS) in the Experiment 2

**Fig. S20** Model of *M. alpina*-induced stress response signaling in wheat. *M. alpina* strongly induces expression of genes in the CIPK and PP2C families, CIPK-PP2C complexes activate *MAPKKK17*, and the activated MAPK cascade induces the expression of the transcription factors MYB36, MYB62, and NAC71 to regulate the expression of drought-responsive genes. However, suppression by *E. nigrum* of expression of the *MAPK6* and *NAC71* genes leads to repression of drought-responsive genes.

**Table S1** Annual precipitation and average monthly precipitation at the Suqian (SQ) and Yangling (YL) planting sites.

See the **Additional file 1**: Table S1 (XLSX).

**Table S2** Primers used for quantitative real-time PCR (qRT-PCR) in this study.

See the **Additional file 1**: Table S2 (XLSX).

**Table S3** Effects of genotypes, planting sites, and niche compartments on the bacterial and fungal communities based on PERMANOVA analysis.

See the **Additional file 1**: Table S3 (XLSX).

**Table S4** Relative abundances of dominant bacterial phyla for YH and CS wheats at the SQ and YL sites

See the **Additional file 1**: Table S4 (XLSX).

**Table S5** Relative abundances of dominant fungal phyla for YH and CS wheats at SQ and YL sites.

See the **Additional file 1**: Table S5 (XLSX).

**Table S6** Drought-responsive bacterial OTUs identified at genus level.

See the **Additional file 1**: Table S6 (XLSX).

**Table S7** Drought-responsive fungal OTUs identified at genus level.

See the **Additional file 1**: Table S7 (XLSX).

**Table S8** The relative abundance of plant pathogenic OTUs at phyla level and at genus level in our samples.

See the **Additional file 1**: Table S8 (XLSX).

**Table S9** Topological characteristics of the microbial interkingdom association networks in the YH and CS sites.

See the **Additional file 1**: Table S9 (XLSX).

**Table S10** Metagenomic sequencing data characteristics for rhizosphere microbiomes from different wheat cultivars at the SQ and YL sites.

See the **Additional file 1**: Table S10 (XLSX).

**Table S11** Root morphology traits of wheat sample under different microbial inoculation treatments in the Experiment 1.

See the **Additional file 1**: Table S11 (XLSX).

**Table S12** Spearman’s Correlation coefficients between rhizosphere microorganisms and root morphological traits.

See the **Additional file 1**: Table S12 (XLSX).

**Table S13** Root morphology traits of China Spring in the Experiment 2

See the **Additional file 1**: Table S13 (XLSX).

**Table S14** Division by k-means clustering of differentially expressed genes (DEGs) into clusters 8.

See the **Additional file 1**: Table S14 (XLSX).

**Table S15** Gene IDs of DEGs involved in responses to abiotic stress and mitogen-activated protein Kinase activity.

See the **Additional file 1**: Table S15 (XLSX).

**Fig. S1** Rhizosphere soil microbiome diversity and distribution patterns. **(a)** Canonical analysis of principal coordinates (CAP) of bacteria and fungi was performed to determine whether there were differences in samples according to the Bray–Curtis dissimilarity matrix. **(b)** Distinct beta-diversity between bacterial and fungal communities was shown by principal coordinate analysis ordinations (PCoA). Abbreviations: Suqian site (SQ), Yangling site (YL), drought-susceptible wheat cultivar Chinese Spring (CS), drought-resistant wheat cultivar Yunhan 618 (YH), bulk soil (B), and rhizosphere soil (T).


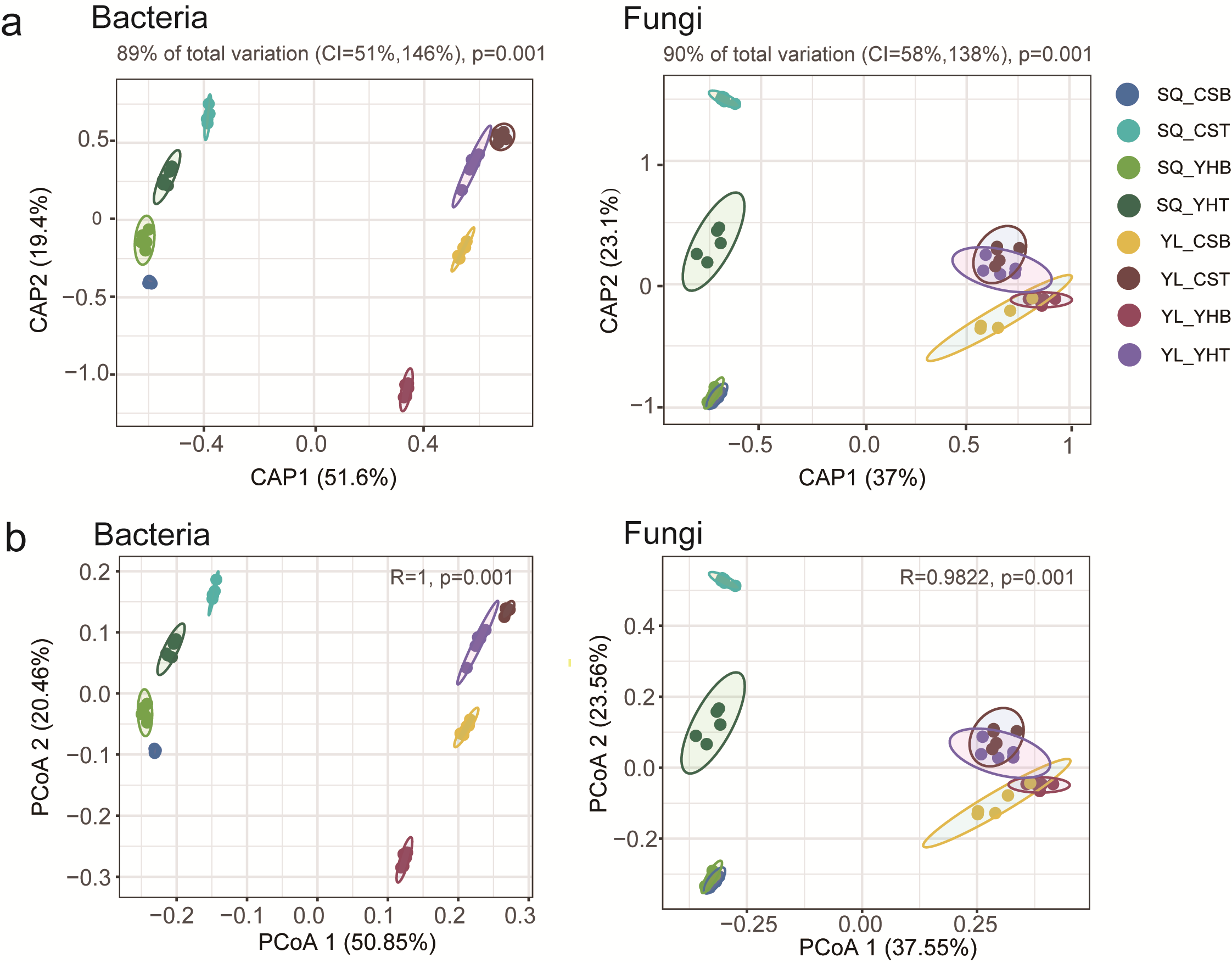


**Fig. S2** Soil microbiome diversity in bulk and rhizosphere soils. (**a)** Results of CAP of bacterial and fungal microbiota in bulk and rhizosphere soils. **(b)** PCoA plots of the bacterial and fungal community structure.


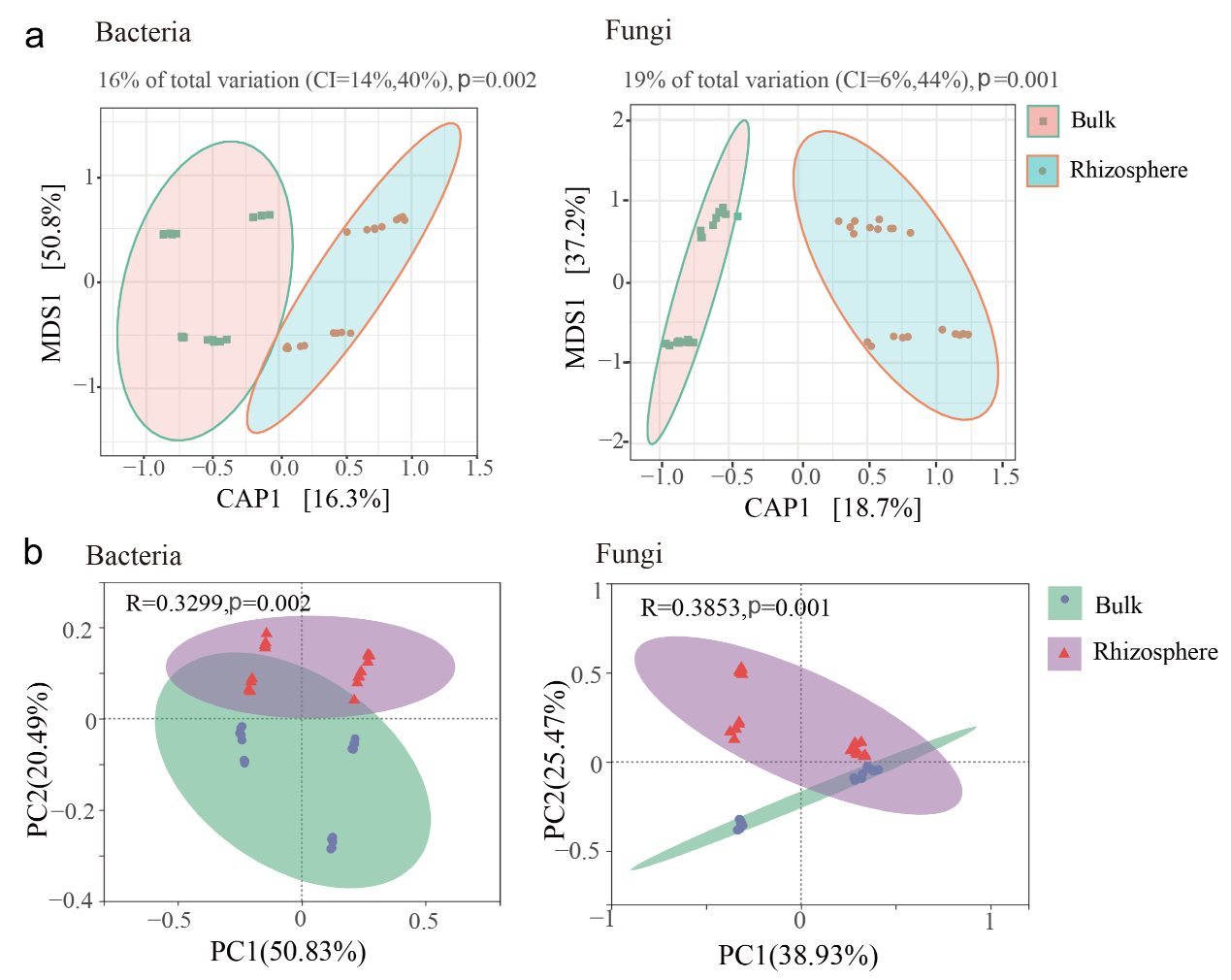


**Fig. S3** Phylogenetic tree for bacterial **(a)** and fungal **(b)** communities at the phylum level showing the hierarchical relationships of operational taxonomic units (OTUs) with an average abundance of greater than 3000 in each sample population (inner circle). The outer rings indicate the abundance and distribution of OTUs in all samples. The values indicate the average abundance of each OTU (log-transformed). The colors represent the OTUs at the phylum level.

**
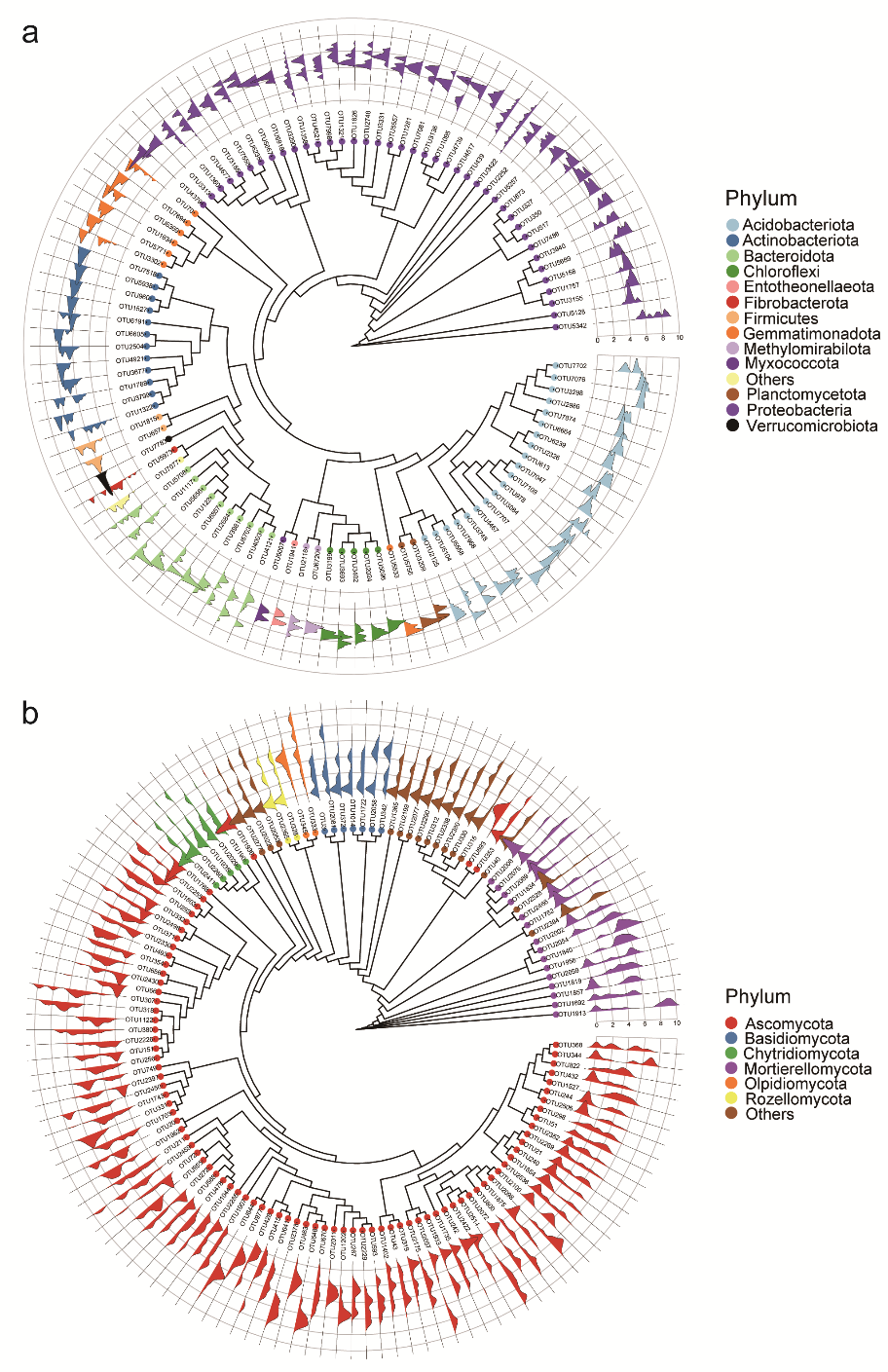
**

**Fig. S4** Differential abundance of bacteria and random forest classification revealing differences in rhizosphere microbial structure. **(a)** and **(b)** Volcano plots visualizing the enrichment and depletion patterns of bacteria in YH in comparison with CS at SQ **(a)** and YL **(b)**. Black dots represent no significant differences in OTUs. Red and blue dots indicate an individual OTU significantly enriched in YH and CS, respectively. **(c)** and **(d)** The influence of YH and CS at SQ **(c)** and YL **(d)** on the 11 most important bacterial taxa in rhizosphere soil was determined by random forest classification. The relative abundance of rhizobacteria differed in accordance with the drought tolerance of the sample. OTUs are arranged along the y-axis in descending order of importance by calculating the Gini coefficient to validate the accuracy of the model. For the abbreviations see Fig. S1.


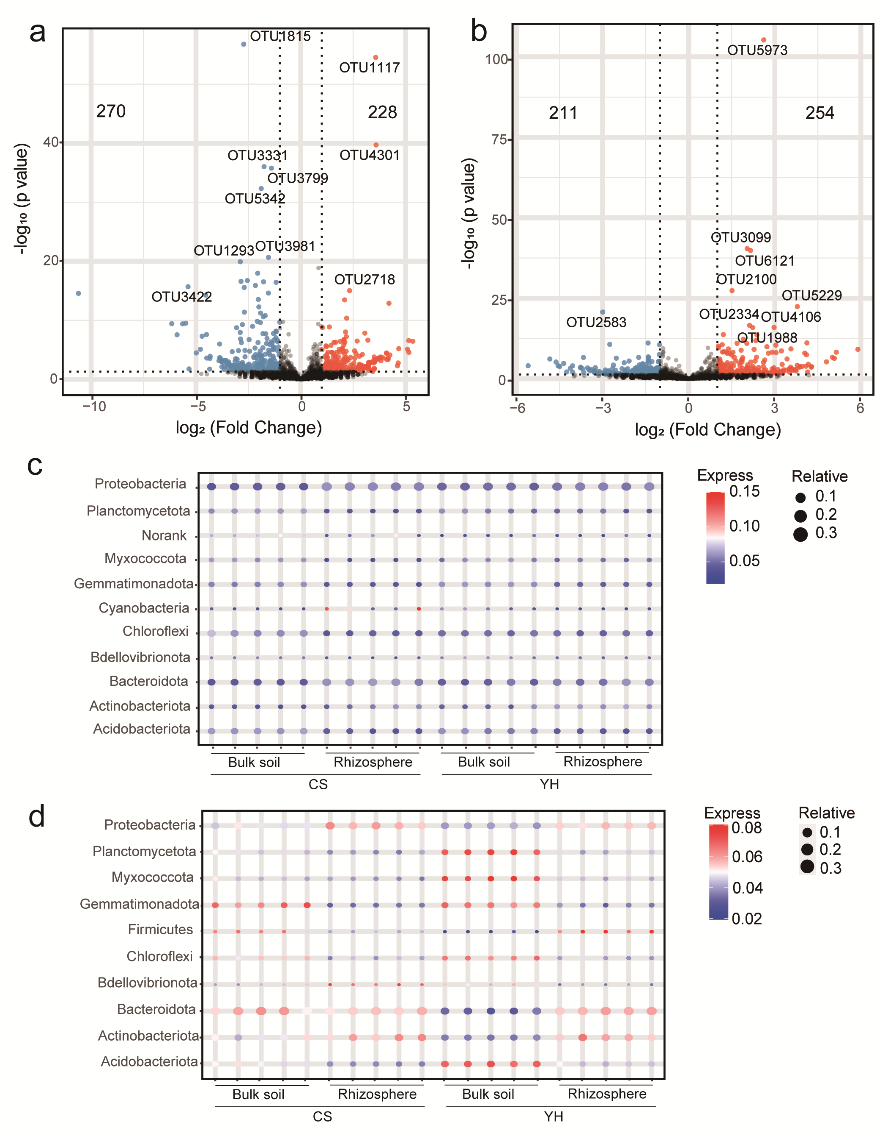


**Fig. S5** Differential abundance of fungi and random forest classification revealing differences in rhizosphere microbial structure. **(a)** and **(b)** Volcano plots visualizing the enrichment and depletion patterns of fungi in YH in comparison with CS at SQ **(a)** and YL **(b)**. **(c)** and **(d)** The influence of YH and CS at SQ **(c)** and YL **(d)** on the five most important fungal taxa in rhizosphere soil was determined by random forest classification.


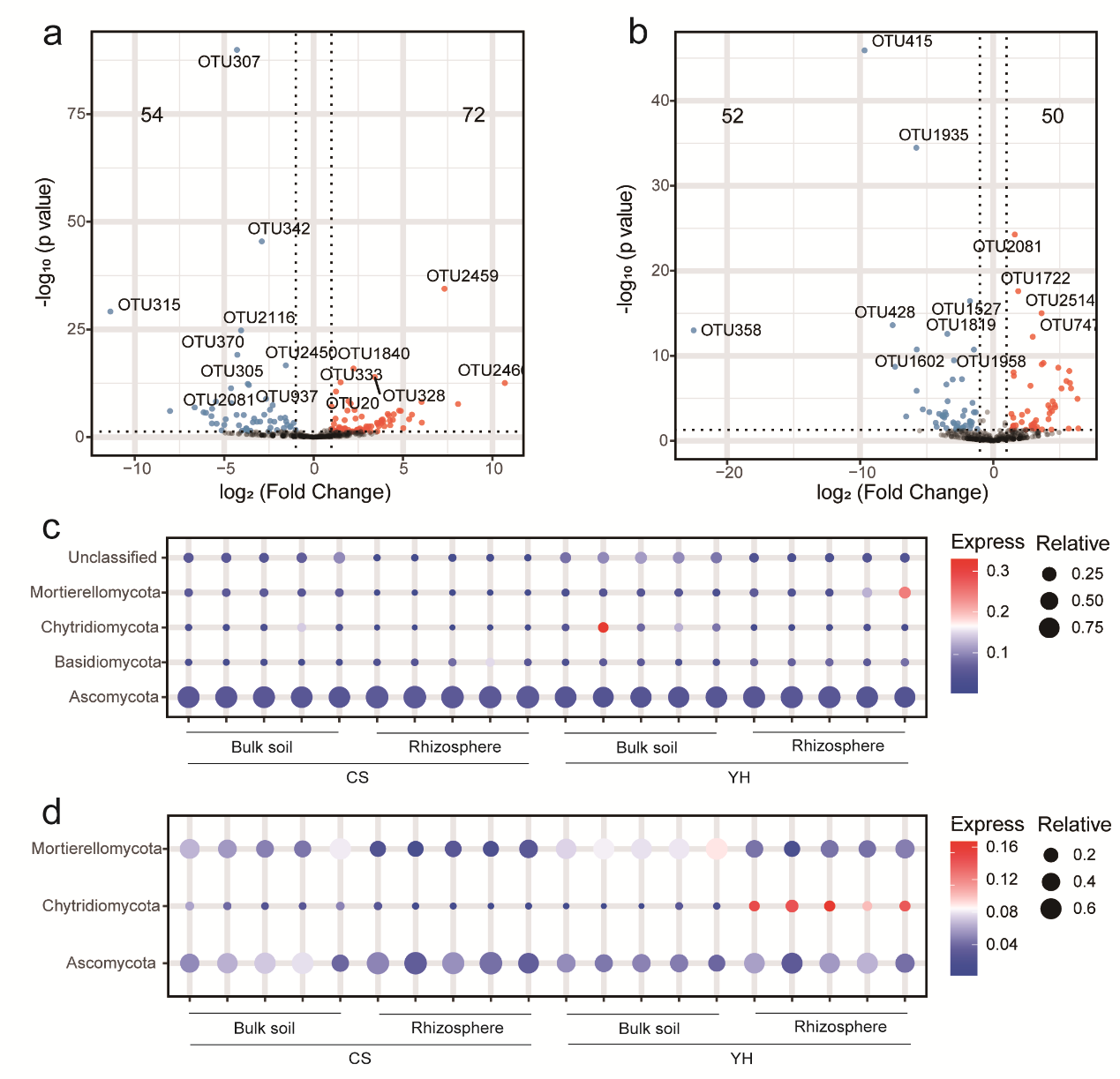


**Fig. S6** Heatmap showing the relative abundances of abundant genera present in drought-responsive bacteria and fungi, which varied between the drought-resistant cultivar and the drought-susceptible cultivar at the two sites. All taxa are indicated according to their significance (Wilcox test, *P* < 0.05) in the different samples. The colors represent the log_2_ fold change (log_2_FC) in relative abundance.

**
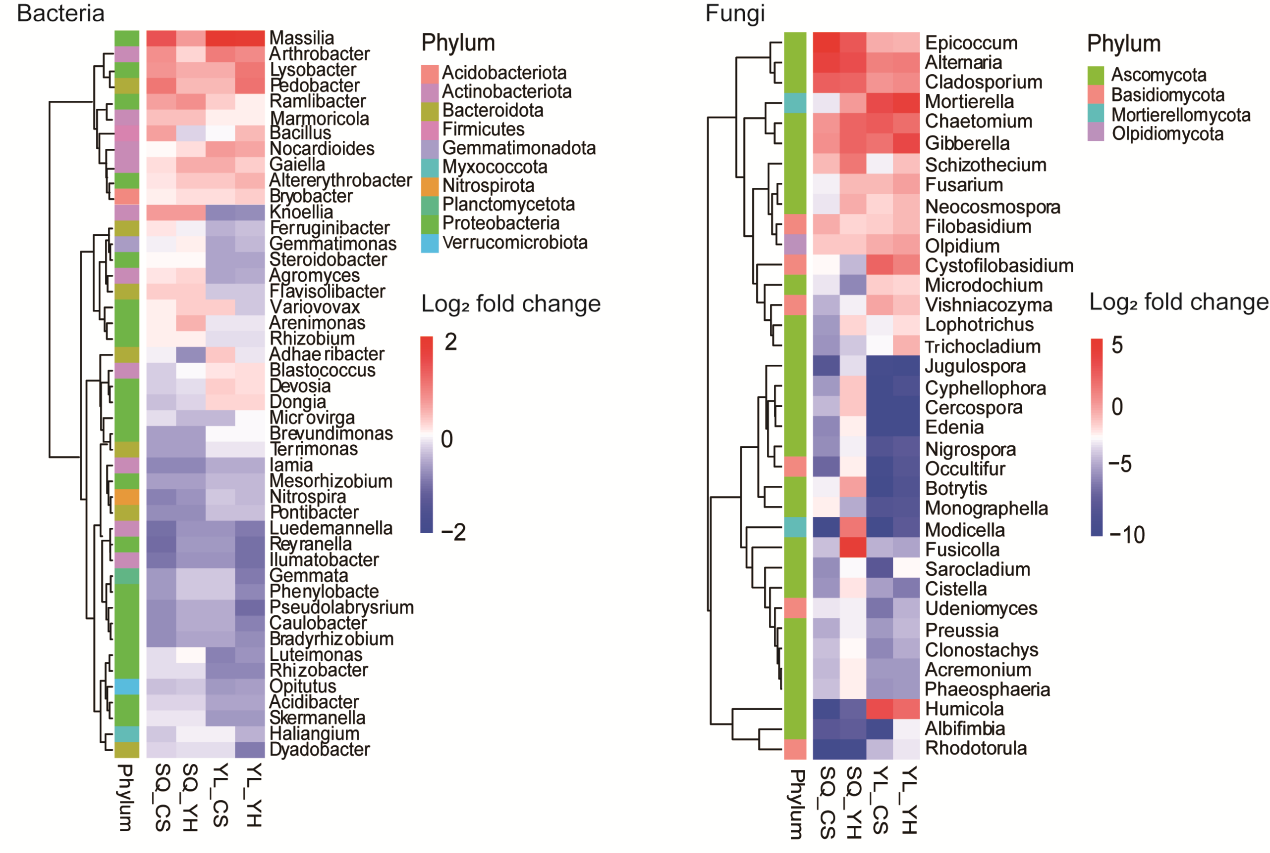
**

**Fig. S7** The pathogenic ASVs included in the fungi phyla Basidiomycota, Blastocladiomycota, and Chytridiomycota for all samples.


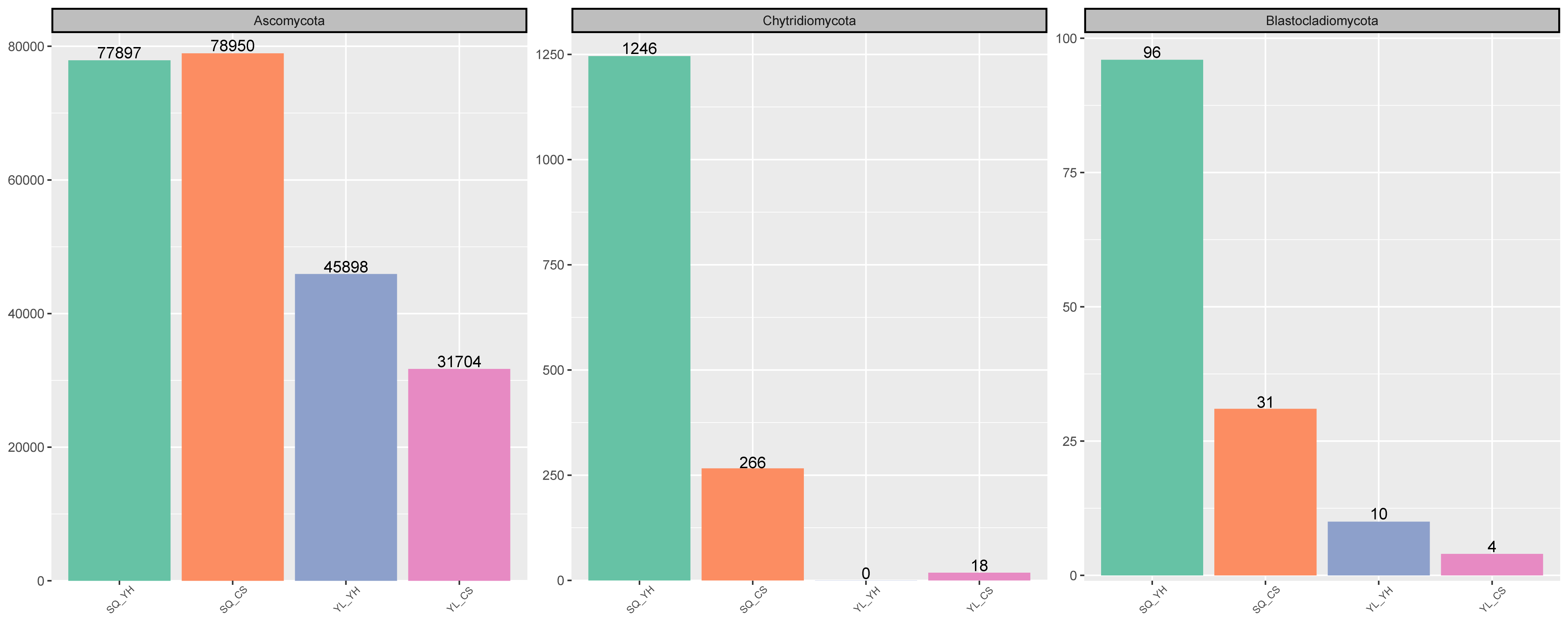


**Fig. S8** PCoA plots of functional traits of the rhizosphere bacterial and fungal communities. **(a)** PCoA plots of functional traits using relative abundances based on Kyoto Encyclopedia of Genes and Genomes (KEGG) pathway level 3, **(b)** orthologous groups of proteins (COG), and **(c)** carbohydrate-active enzymes (CAZyome).


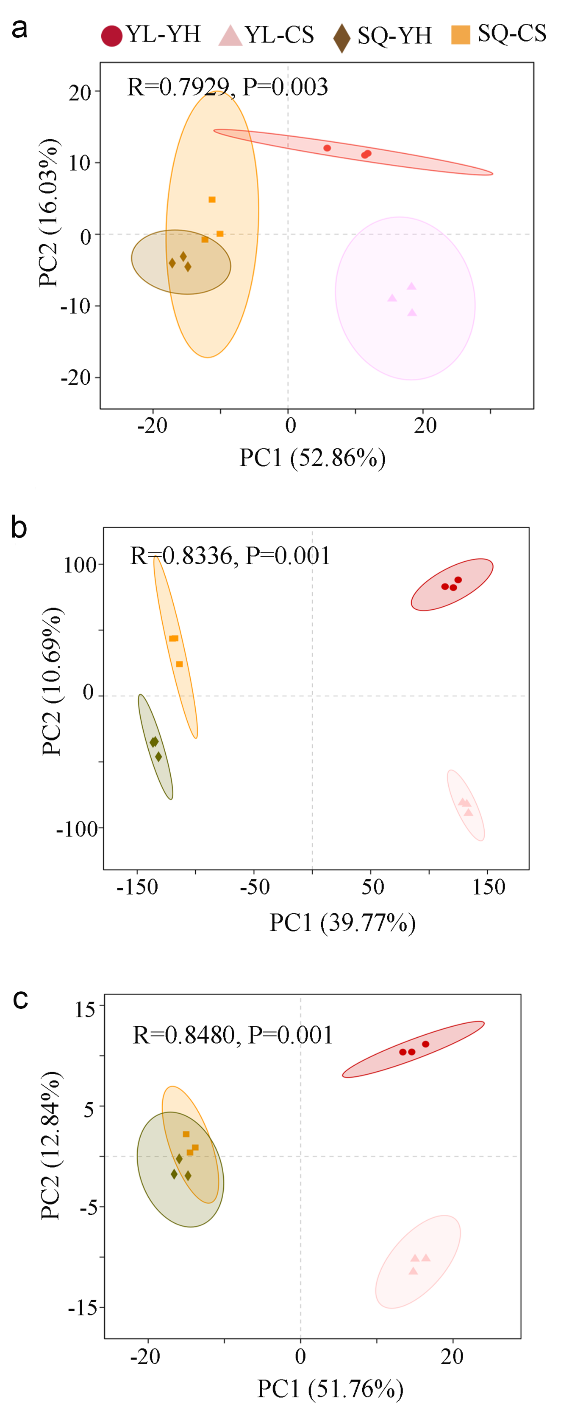


**Fig. S9** Diversity indices for functional categories, including KEGG pathway level 3 **(a)**, CAZyome **(b)**, and COG **(c)**, for each sample from rhizosphere soil. A difference in the letters on top of each boxplot suggests a statistically significant difference according to one-way analysis of variance (*P* < 0.05).


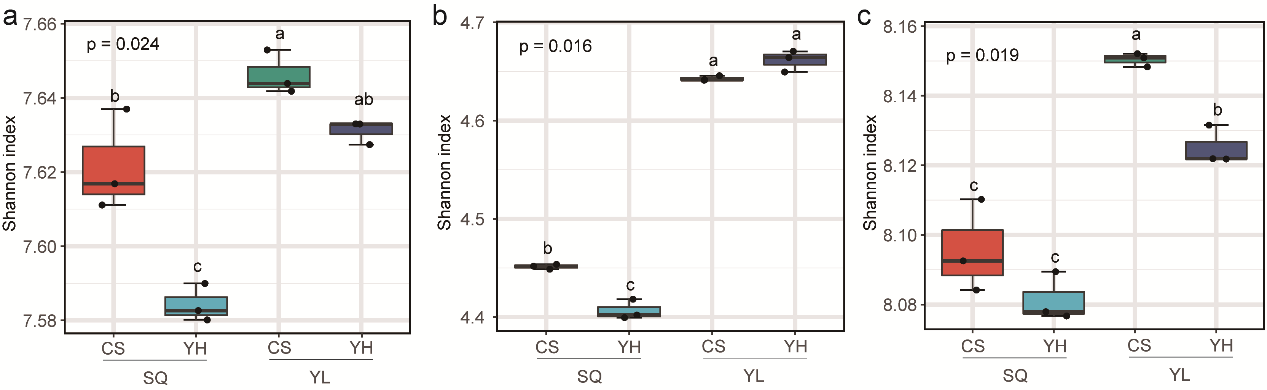


**Fig. S10** Volcano plots visualizing the KEGG Orthology (KO) pathways and functional categories. **(a)** and **(b)** Volcano plots visualizing the KO pathways that were significantly enriched or depleted in the drought-resistant cultivar in comparison with the drought-susceptible cultivar at the YL and SQ sites. DI and DSI represent the depletion index and dissimilarity index, respectively. Grey dots indicate that there were no significant differences in OTUs. Dark blue and yellow dots indicate an individual OTU significantly enriched in the drought-resistant cultivar and the drought-susceptible cultivar, respectively. **(c)** and **(d)** Volcano plots visualizing the KO functional categories that were significantly enriched or depleted in the drought-resistant cultivar in comparison with the drought-susceptible cultivar at the YL and SQ sites.


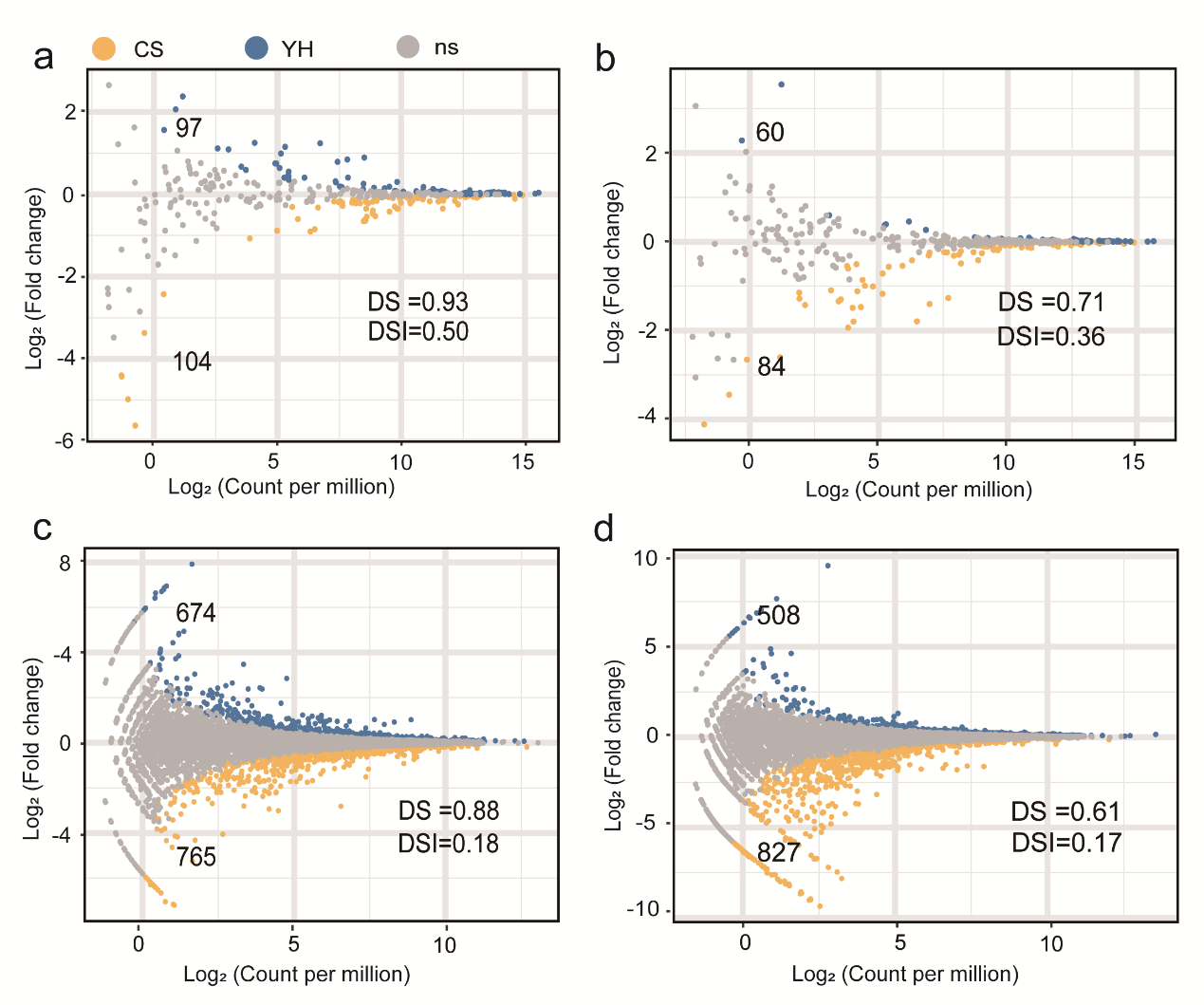


**Fig. S11** Functional profile based on KEGG pathway level 3 **(a)**, CAZyome **(b)**, and COG **(c)** of top OTUs in four samples.


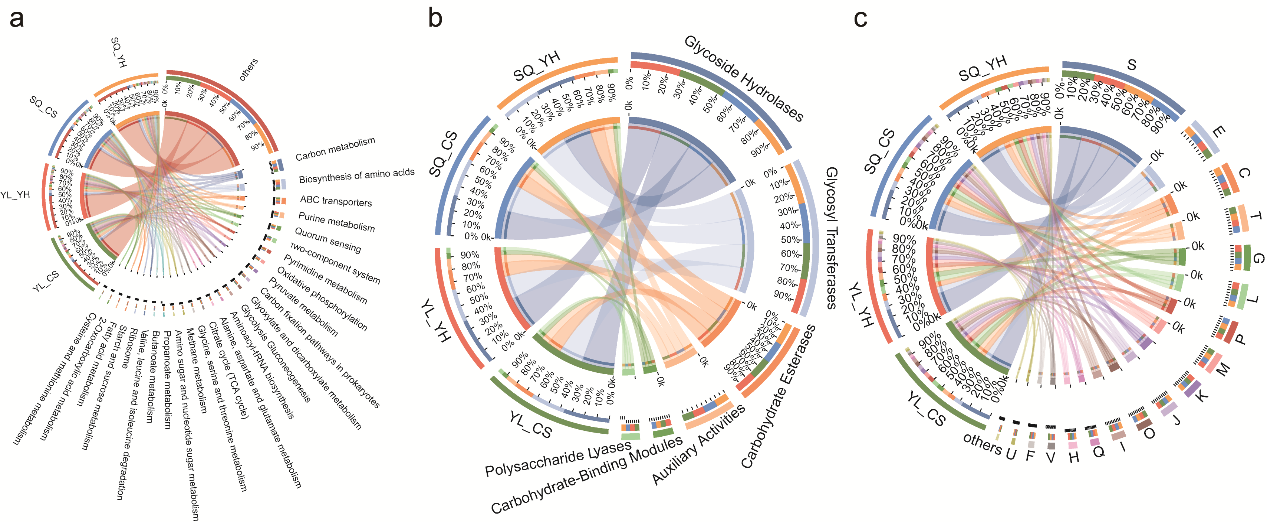


**Fig. S12** Functional profiles of rhizosphere microbiomes based on KO. KO functional categories and pathways that were significantly enriched in the drought-susceptible cultivar and drought-resistant cultivar at the YL **(a)** and SQ **(b)** sites. Red and dark blue colors represent significantly enriched KO pathways in YH and CS wheats, respectively.


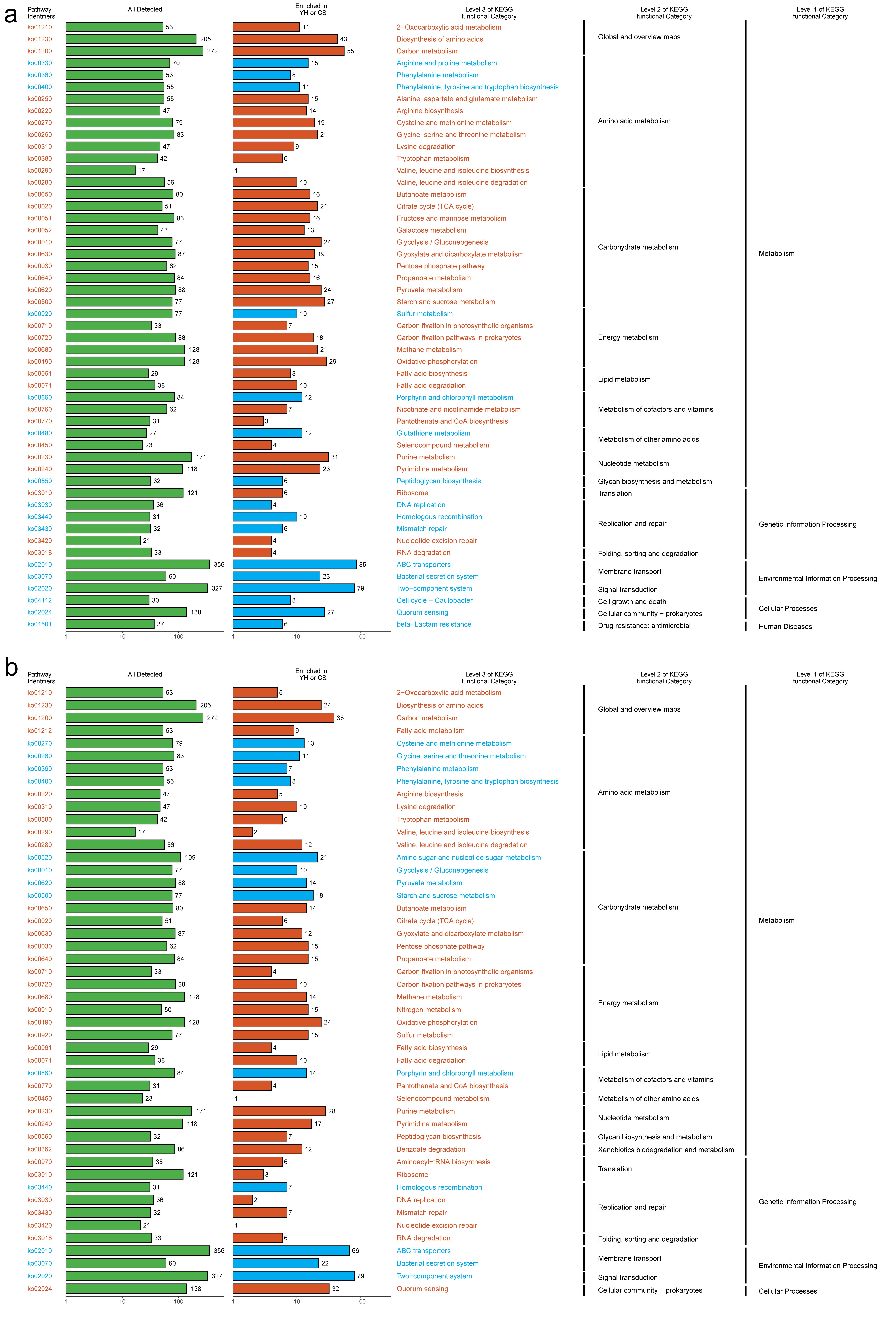


**Fig. S13** Relative abundance of functional genes associated with drought responses that varied between the drought-resistant cultivar and the drought-susceptible cultivar.


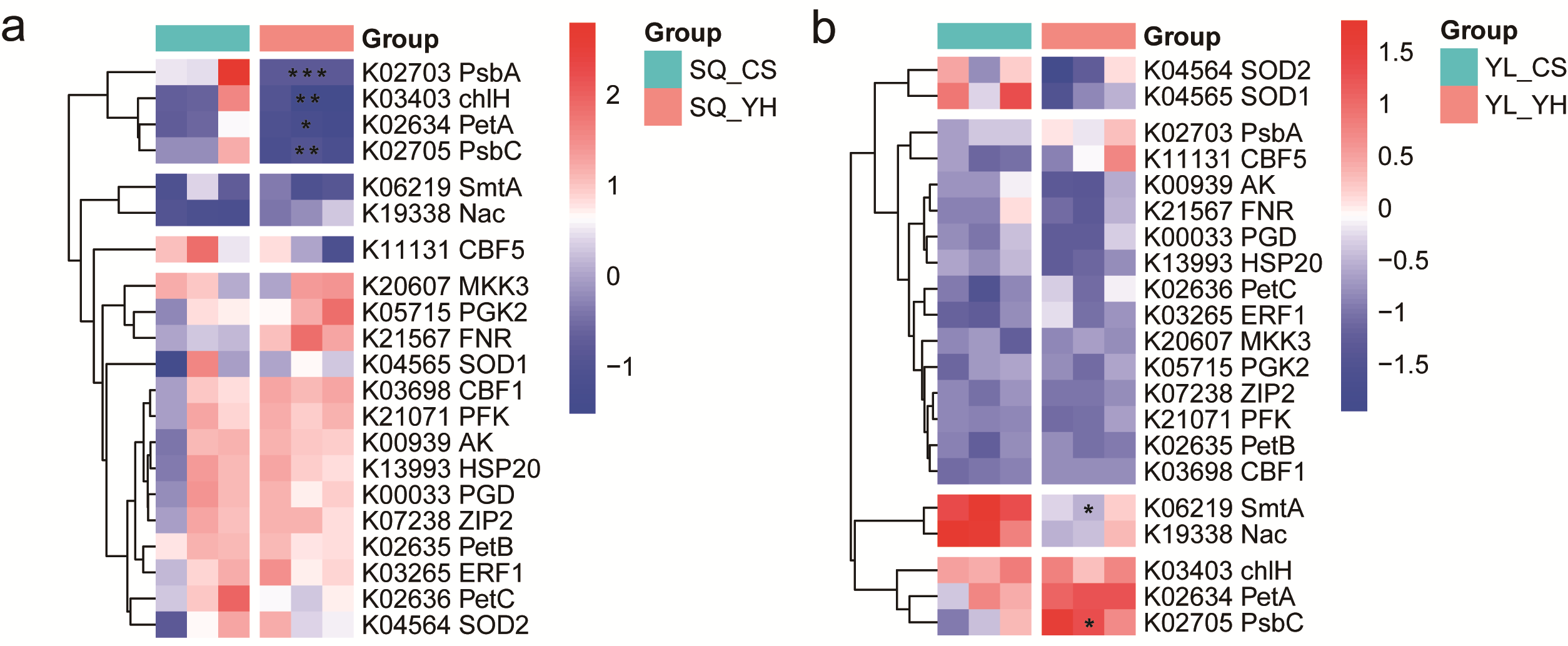


**Fig. S14** Variations in rhizosphere microbial communities explaining differences in wheat root system architecture. Keystone bacterial and fungal OTUs significantly correlated with root system architecture in wheat grown in Hoagland nutrient solution treated with microbial suspensions (log_2_FC > 2, *P* < 0.05). Heatmap displaying the top 30 bacterial OTUs and 30 fungal OTUs on the basis of relative abundance distributions. Blue in the top panel indicates that the OTU was enriched in CS, and orange indicates that the OTU was enriched in YH. **p* < 0.05, ***p* < 0.01, and ****p* < 0.001.


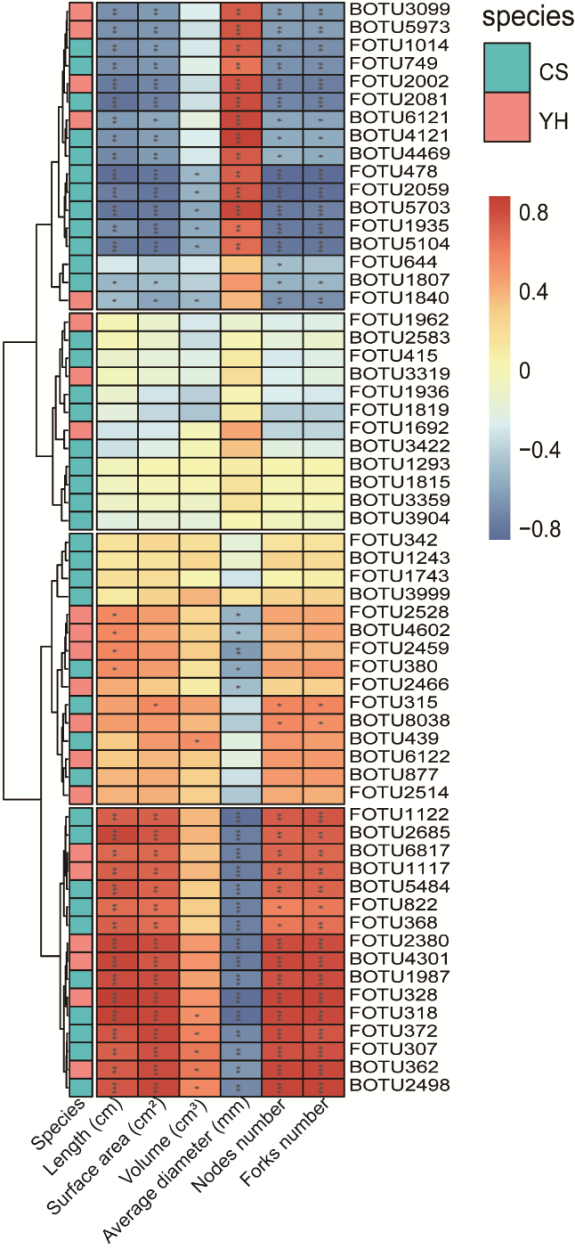


**Fig. S15** DEGs in *M. alpina*, *E. nigrum*, and SynCom-2 and results of KO functional analyses. Volcano plots of DEGs in wheats grown in Hoagland nutrient solution with or without fungal inoculation under drought stress. The threshold for statistically significant differential expression was fold change > 1 and q-value < 0.05. Upregulated genes are shown in red, and downregulated genes are shown in blue.

**
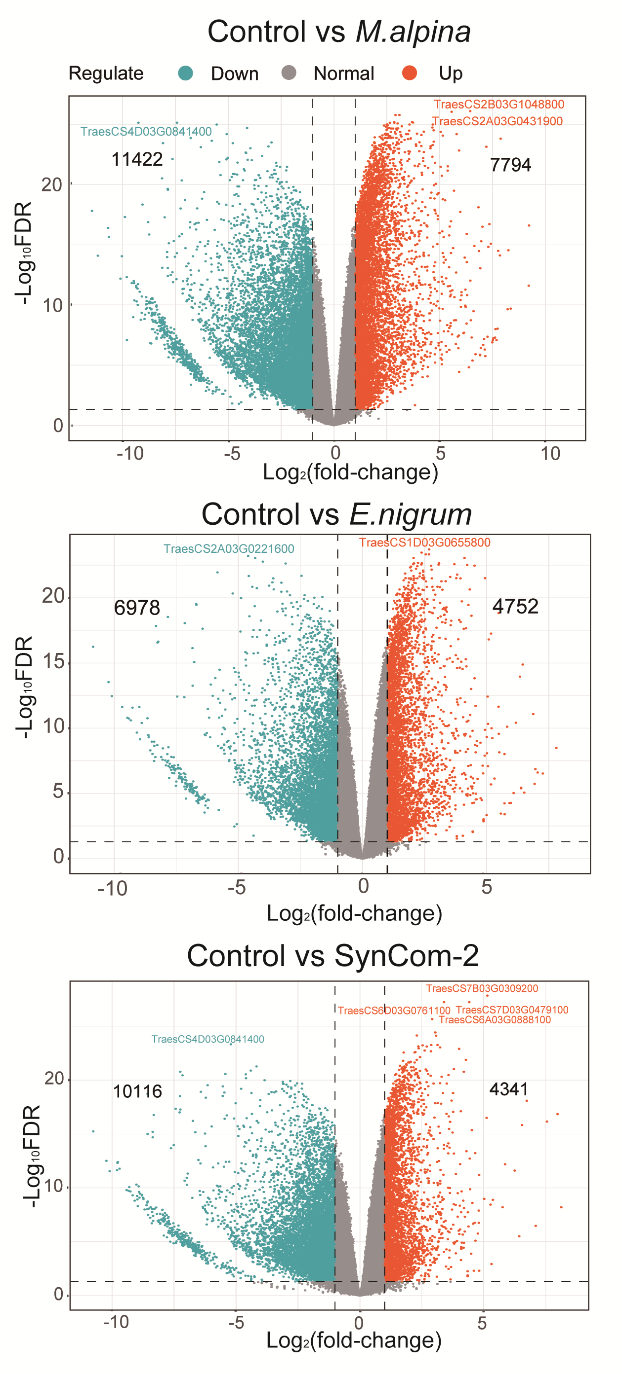
**

**Fig. S16** Division of all DEGs into eight clusters by k-means clustering analysis.

**
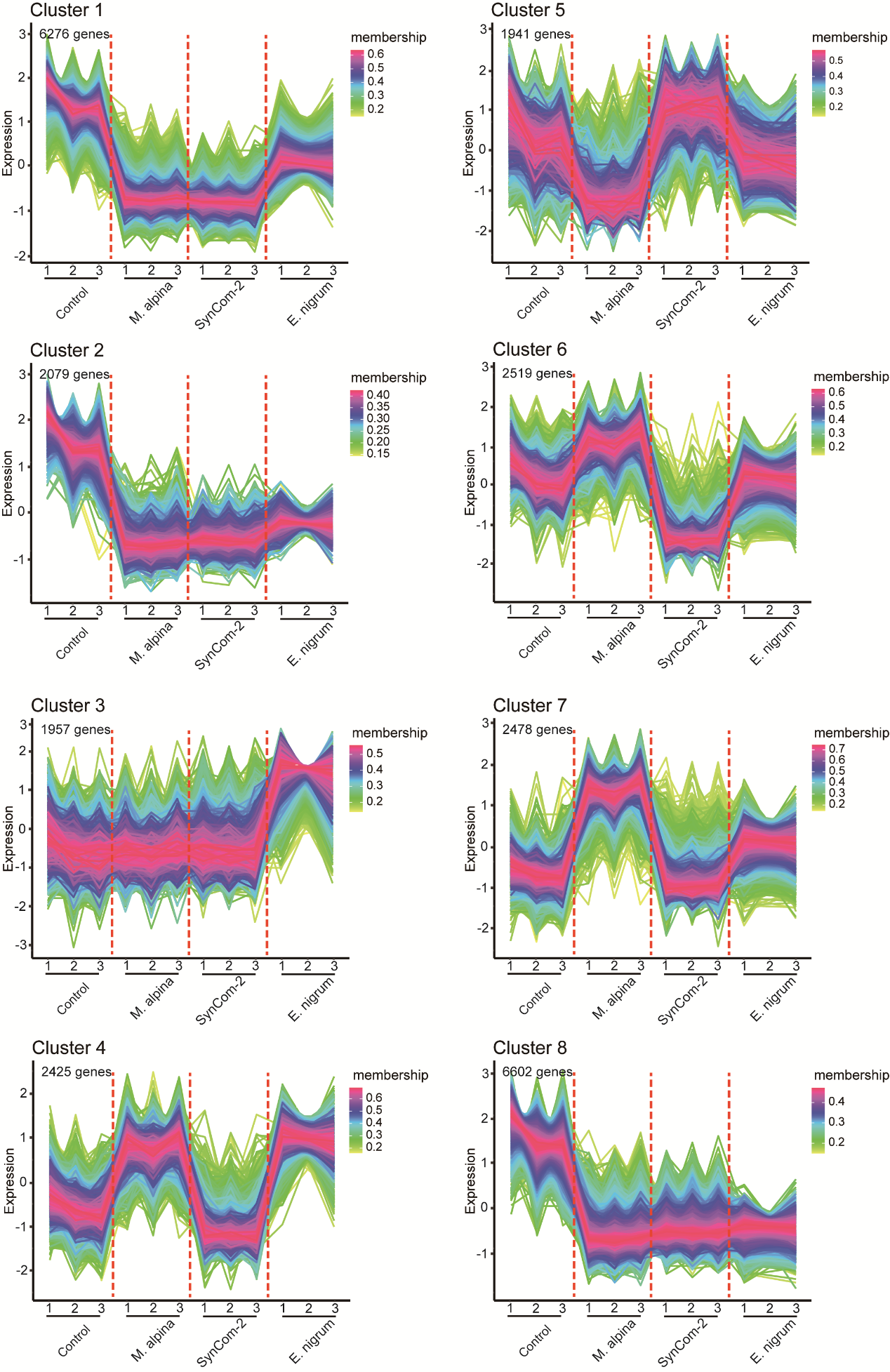
**

**Fig. S17** Relative expression levels (2^−ΔΔCT^) of drought response-related genes obtained by qRT-PCR analysis.


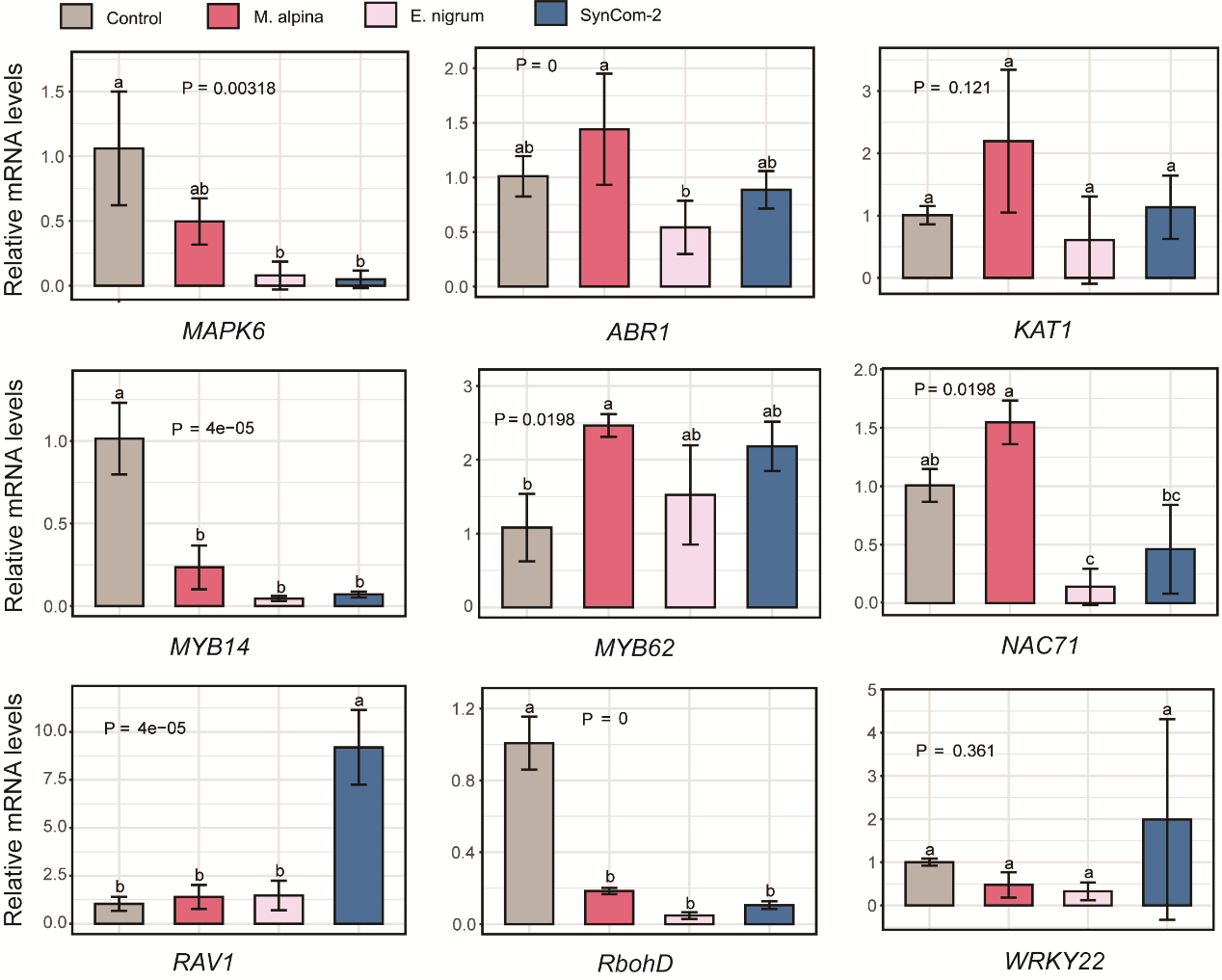


**Fig. S18** Relative expression levels (2^−ΔΔCT^) of root development-related genes obtained by RNA-seq analysis.


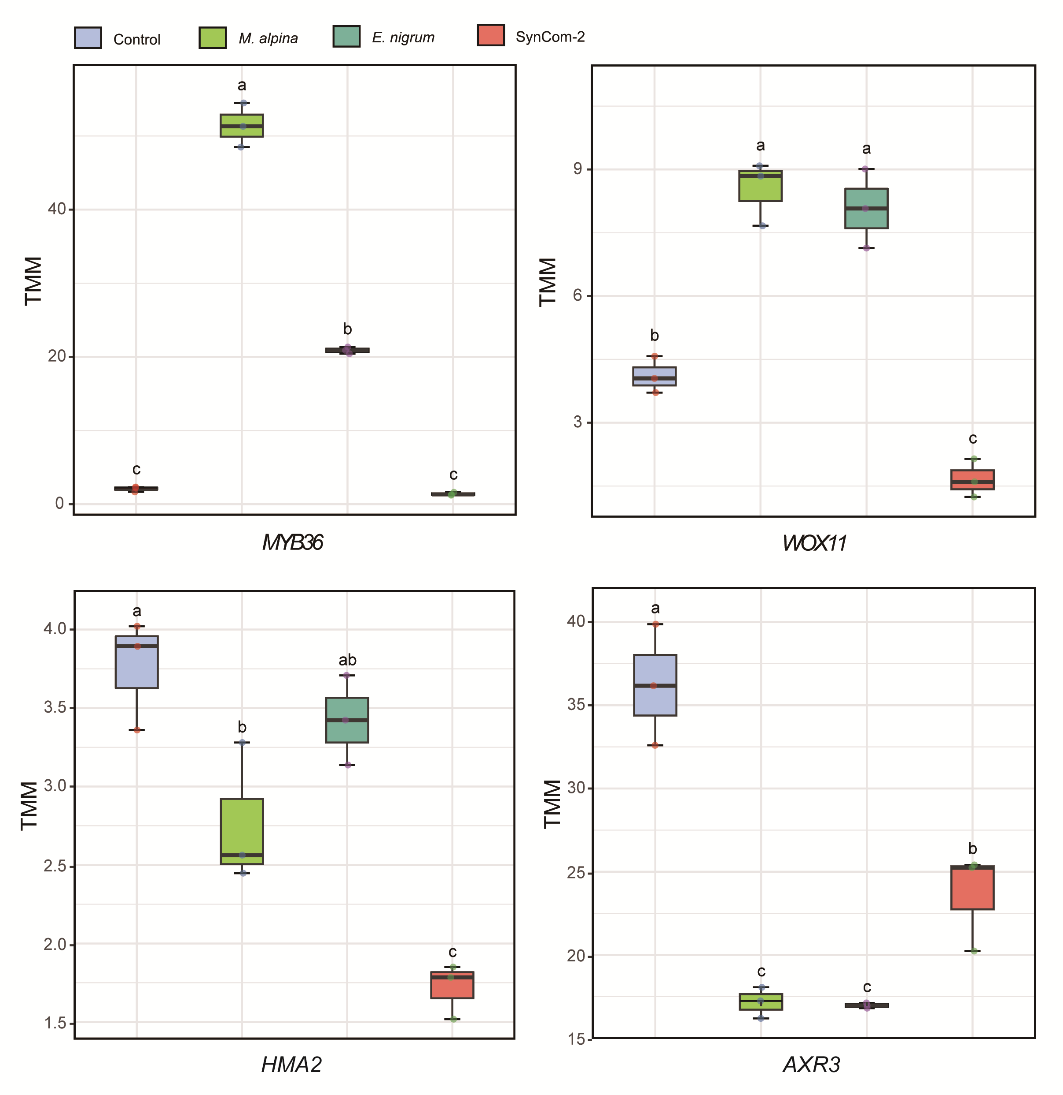


**Fig. S19** Root-related parameters of China Spring in the Experiment 2


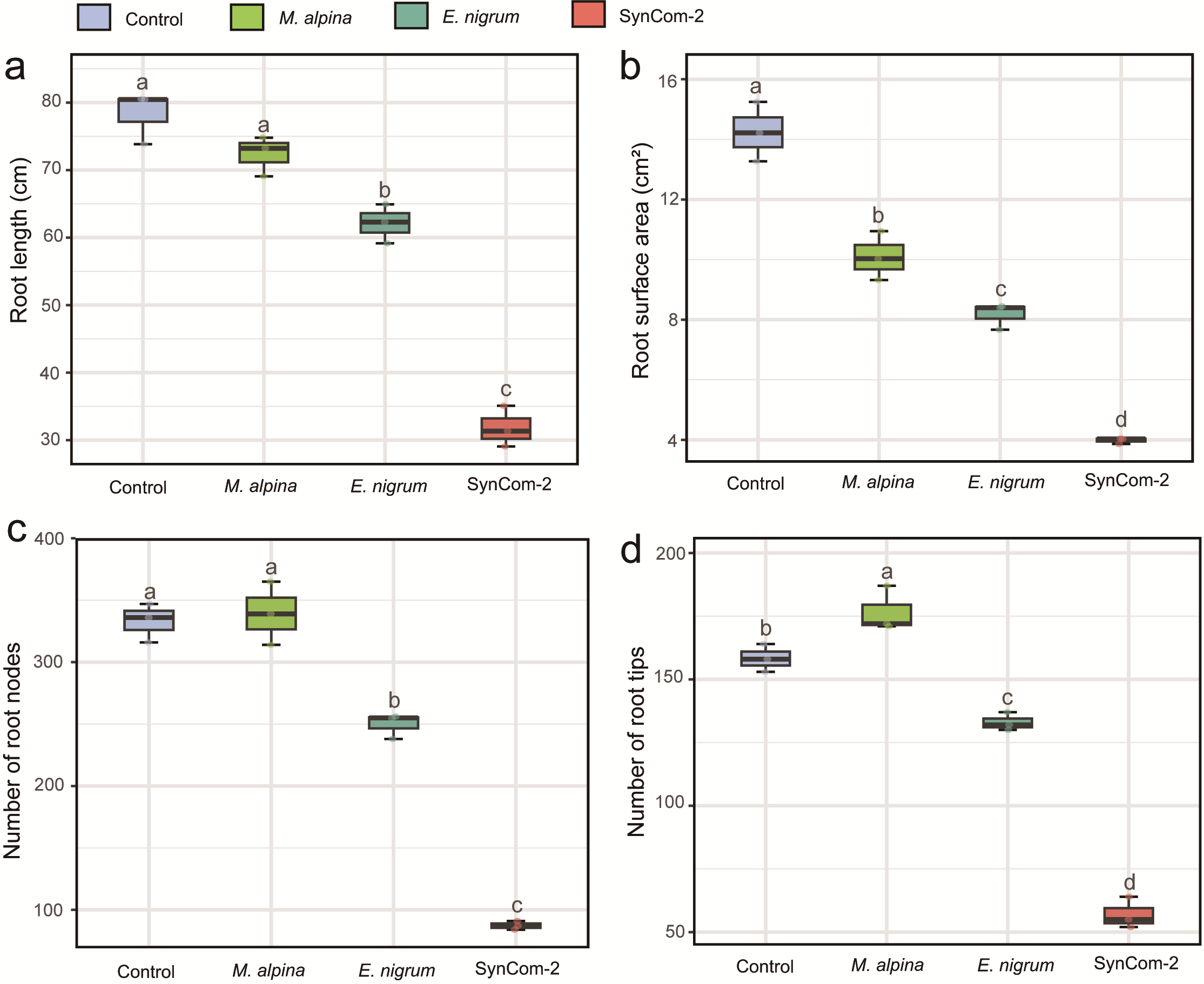


**Fig. S20** Model of *M. alpina*-induced stress response signaling in wheat. *M. alpina* strongly induces expression of genes in the CIPK and PP2C families, CIPK-PP2C complexes activate *MAPKKK17*, and the activated MAPK cascade induces the expression of the transcription factors MYB36, MYB62, and NAC71 to regulate the expression of drought-responsive genes. However, suppression by *E. nigrum* of expression of the *MAPK6* and *NAC71* genes leads to repression of drought-responsive genes.


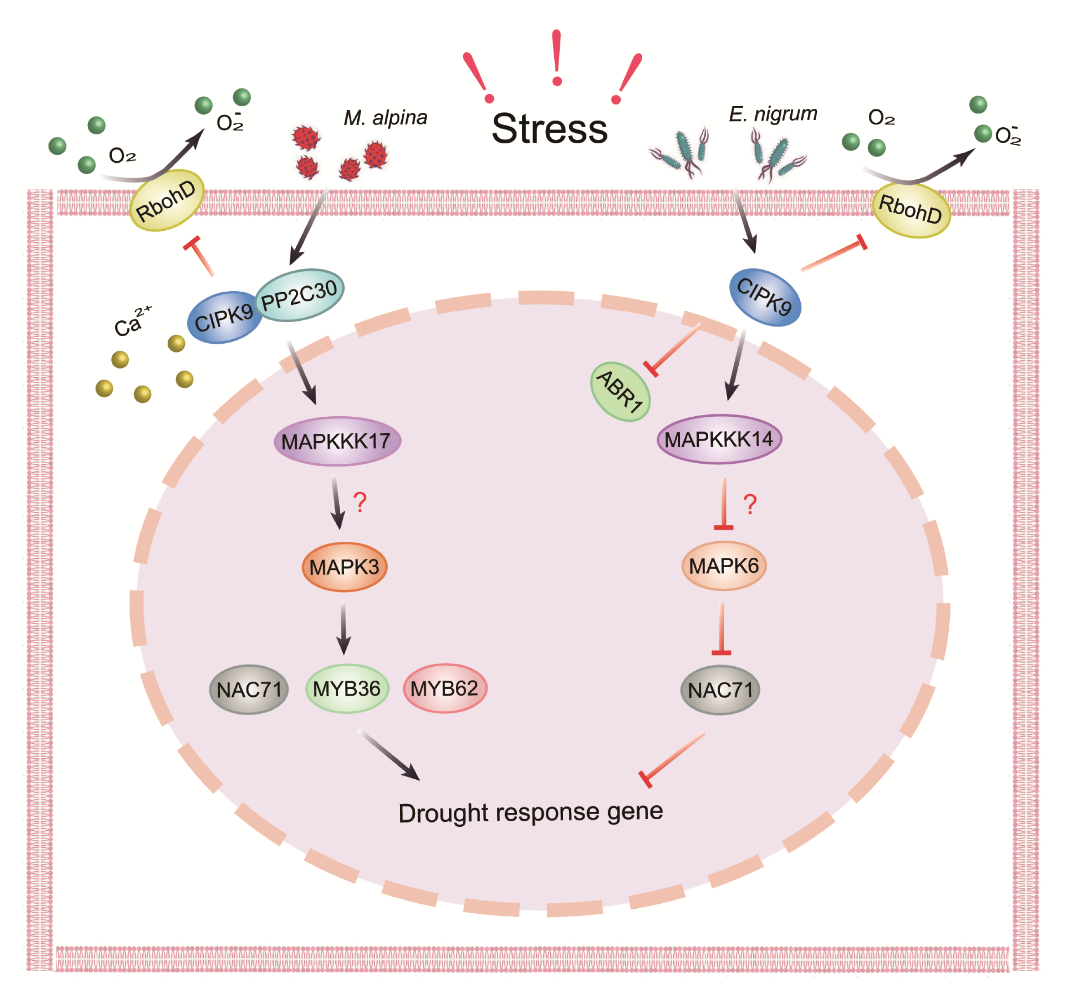

Supplement: Supplementary file 2 — Additional file 1: Table S1. Annual precipitation and average monthly precipitation at the Suqian (SQ) and Yangling (YL) planting sites. Table S2. Primers used for quantitative real-time PCR (qRT-PCR) in this study. Table S3. Effects of genotypes, planting sites, and niche compartments on the bacterial and fungal communities based on PERMANOVA analysis. Table S4. Relative abundances of dominant bacterial phyla for YH and CS wheats at the SQ and YL sites. Table S5. Relative abundances of dominant fungal phyla for YH and CS wheats at SQ and YL sites. Table S6. Relative abundances of drought-responsive bacterial OTUs at genus level. Table S7. Relative abundances of drought-responsive fungal OTUs at genus level. Table S8. The absolute abundance of plant pathogenic OTUs at phyla and genus level. Table S9. Topological characteristics of the microbial interkingdom association networks in the YH and CS sites. Table S10. Metagenomics sequencing data characteristics for rhizosphere microbiomes from different wheat cultivars at the SQ and YL sites. Table S11. Root morphology traits of wheat sample under different microbial inoculation treatments in the Experiment 1. Table S12. Spearman’s Correlation coefficients between rhizosphere microorganisms and root morphological traits. Table S13. Root morphology traits of China Spring (CS) in the Experiment 2. Table S14. Division by k-means clustering of differentially expressed genes (DEGs) into clusters 8. Table S15. Gene IDs of DEGs involved in responses to abiotic stress and mitogen-activated protein Kinase activity. Fig. S1. Rhizosphere soil microbiome diversity and distribution patterns. (a) Canonical analysis of principal coordinates (CAP) of bacteria and fungi was performed to determine whether there were differences in samples according to the Bray–Curtis dissimilarity matrix. (b) Distinct beta-diversity between bacterial and fungal communities was shown by principal coordinate analysis ordinations (PCoA). Abbreviations: Suqia [file 40168_2024_1770_MOESM1_ESM.zip › Supplementary_datas_0112.docx]
